# *Roseburia intestinalis* ameliorates adolescent depression via GPR43‑dependent Treg cell expansion and suppression of neuroinflammation

**DOI:** 10.1186/s12974-026-03755-w

**Published:** 2026-03-03

**Authors:** Jing Liu, Le Fu, Zhiru Tang, Xiaozhu Li, A Yiguzaili Manglike, Xi Wang, Hui Mo, Gang Liu, Lei Jiang, Rong Gao, Jun Wang

**Affiliations:** 1 Department of Hygienic Analysis and Detection, the Key Laboratory of Modern Toxicology, Ministry of Education, School of Public Health, Nanjing Medical University, Nanjing, 211166 China; 2 Department of Toxicology, Center for Global Health, the Key Laboratory of Modern Toxicology, Ministry of Education, School of Public Health, Nanjing Medical University, Nanjing, 211166 China; 3 Department of Psychology, Nanjing Hospital of Traditional Chinese Medicine, Nanjing, 210029 China; 4 Department of Psychiatry, Affiliated Nanjing Brain Hospital, Nanjing Medical University, Nanjing, 210029 China; 5 Department of Emergency Medicine, the First Affiliated Hospital of Nanjing Medical University, 300 Guangzhou Road, Nanjing, Jiangsu 210029 China

**Keywords:** Gut microbiota, Adolescent depression, Roseburia intestinalis, Butyric acid, Treg cells

## Abstract

Adolescent depression has rapidly emerged as a critical public health concern, driven by its soaring prevalence, profound functional impairment, and heightened suicide risk. However, the underlying mechanisms remain poorly understood. A growing body of evidence highlights the gut microbiota-brain axis as a key modulator of host emotional function, acting through interconnected immune, metabolic, and neural pathways. In the current work, a clinical study involving first-diagnosed adolescent depression patients was conducted and significant reductions in *Roseburia intestinalis* (*R.i.*) and its metabolite butyrate were identified. Therefore, we hypothesized that alterations in *R.i.* and butyrate may be involved in adolescent depression and may serve as novel therapeutic strategy. Intriguingly, transplantation of *R.i.* obviously retarded the chronic restraint stress (CRS)-induced depression in adolescent mice. Critically, this protective effect was completely abrogated by blocking the G protein-coupled receptor 43 (GPR43). Mechanistically, we delineated a novel “peripheral-central” regulatory cascade: *R.i.* robustly promoted the expansion of peripheral CD25⁺Foxp3⁺ regulatory T (Treg) cells, alleviating pathological inflammation in both of the colon (peripheral) and brain (central). Reciprocally, depletion of Treg cells completely abolished not only *R.i.*’s antidepressant effects but also its anti-inflammatory actions in the colon and brain in vivo. The in vitro study revealed that either the *R.i.* conditioned or heated culture medium obviously alleviated the LPS+corticosterone induced neuroinflammation in microglial cells, shifting reactive state toward a homeostatic state. Collectively, our study for the first time reveals that *R.i.* ameliorates adolescent depression-like changes through a GPR43-dependent, Treg-mediated mechanism. Given the unmet clinical need for clinical diagnosis and therapy of adolescent depression, the present study provided the potential translational values for the novel treatments, such as transplantation of *R.i.* or butyrate supplement, which represent promising therapeutic strategies.

**Supplementary Information:**

The online version contains supplementary material available at 10.1186/s12974-026-03755-w.

## Introduction

Depression is a highly prevalent and recurrent mental disorder, with a growing incidence rate worldwide [1]. Globally, over 350 million people are affected by depression, and adolescents represent one of the high-risk groups. The prevalence of depression among adolescents ranges between 4% and 8%, with half of all first depressive episodes occurring during adolescence. Depression at this critical developmental stage can cause severe impairment of both physical and mental health, and may lead to long-term disability or even death [2]. Furthermore, adolescent depression often persists into adulthood, exerting far-reaching adverse impacts on key domains of life, including academic performance, social functioning, and occupational outcomes [3]. Notably, depression is significantly more prevalent among female adolescents, with an incidence 1.5 to 3 folds higher than that in males [4, 5]. Despite being recognized as a major global public health challenge, diagnosing and treating depression remain clinically difficult, primarily due to its complex etiology, substantial interindividual variability, as well as self-stigmatization. Therefore, a thorough understanding of the underlying mechanisms of adolescent depression is essential for enhancing the efficacy of current treatments. In recent years, the microbiota-gut-brain (MGB) axis has gained increasing attention for its critical roles in the pathogenesis of depression [6]. The bidirectional communication system operates through neural, immune, and endocrine pathways. As an integrative regulatory system, it exerts its functions through mechanisms involving neuroendocrine, neuroimmune, and neuroanatomy, etc [7]. Notably, the gut microbiota produces a range of neuroactive compounds, including neurotransmitters and short-chain fatty acids (SCFAs), and modulates brain function through endocrine signaling. Epidemiological studies have revealed significant alterations in the gut microbiota of individuals with depression, suggesting that gut dysbiosis may be a risk factor for the disorder [8]. Depressed patients exhibit reduced microbial diversity and abundance, along with compositional shifts-such as a decrease in *Firmicutes* and relative increases in *Proteobacteria* [9, 10]. Of all, *Roseburia intestinalis* (*R.i.*), a specific commensal bacterium, shows reduced abundance in depressed patients and may play an important role in the pathogenesis of depression. Alterations in *R.i.* have been linked to multiple diseases, including inflammatory bowel disease and type 2 diabetes [11]. Evidence from existing studies indicates that the abundance of *R.i.* is significantly reduced in individuals with depression [12], whereas it is highly abundant in healthy individuals. Our preliminary study has demonstrated that the abundance of *R.i.* was significantly decreased in patients with depression relative to healthy controls, with an area under the receiver operating characteristic (ROC) curve (AUC) of 0.7333, a value indicative of favorable sensitivity and specificity [13]. These results imply that *R.i.* may hold considerable predictive potential in the development and treatment of depression.

*R.i.* produces SCFAs [14, 15], particularly butyrate, playing vital roles in maintaining intestinal barrier function, immune regulation and neural processes [16]. Butyrate enhances intestinal epithelial barrier integrity by upregulating tight junction proteins and reduces intestinal inflammation. Moreover, it modulates the immune system through two key mechanisms: inhibiting histone deacetylase (HDAC) and activating free fatty acid receptor 2 (FFAR2), which collectively modulate the differentiation of regulatory T (Treg) cells, thereby suppressing excessive inflammation [17]. Importantly, butyrate can cross the blood-brain barrier and act directly on microglia and astrocytes [18]. Similar to its protective effects in the gut, butyrate also contributes to the maintenance of blood-brain barrier integrity, thereby blocking the uncontrolled entry of peripheral inflammatory cells and mediators into the brain parenchyma [19–21]. Therapeutically, butyrate exhibits notable anti-inflammatory potential and can modulate mood and behavior through two key pathways: activation of the vagal nerve and regulation of the hypothalamic-pituitary-adrenal (HPA) axis. Additionally, it exerts direct neuroprotective effects in the central nervous system (CNS) by suppressing nuclear factor-κB (NF-κB) signaling in microglia. Of note, butyrate also influences the brain’s immune environment via peripheral immunomodulation [22, 23], however, the role of the immune system, particularly the peripheral immune system, which acts as a key bridge between the gut and brain, remains a “black box” and is still poorly defined.

Therefore, in the present study, we deciphered the roles of the peripheral immune system, particularly Treg cells in the anti-depressant effects mediated by *R.i.*, moreover, in this context, the effects of Treg cells on regulating microglial activation, cytokine secretion, and neurogenesis were also investigated.

## Methods and materials

**Fig. 1:**
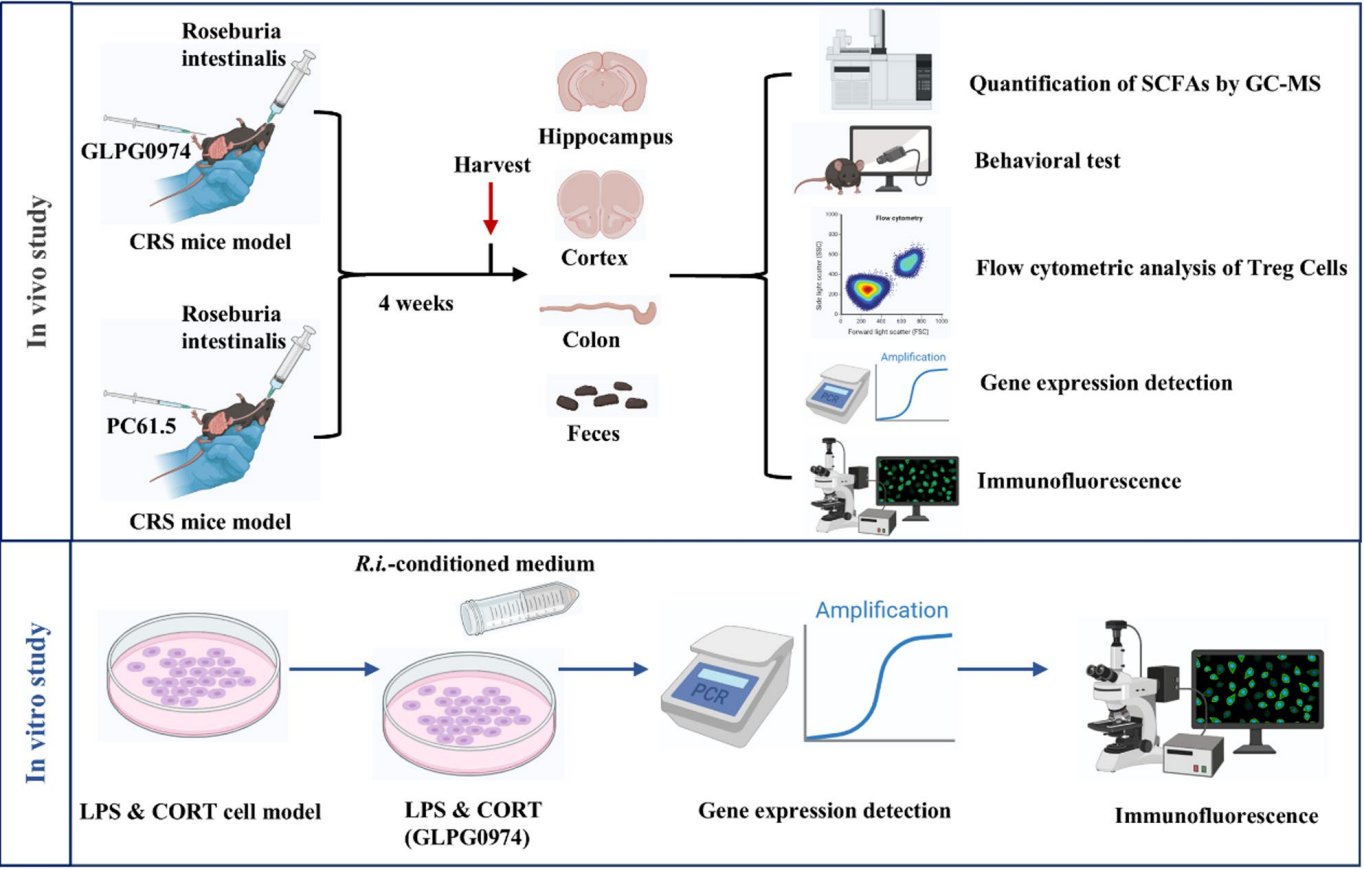
Schematic illustration of the experimental flow in vivo and in vitro experiments. In vivo study, a chronic restraint stress (CRS) mouse model was established and subjected to intervention with *R.i.*, followed by separate administration of an Free Fatty Acid Receptor 2 (FFAR2) inhibitor (GLPG0974) or a CD25-neutralizing antibody (Anti-mouse CD25 Antibody, PC61.5). Afterward, the inflammatory profiles, levels of short-chain fatty acids (SCFAs), immune and neural parameters in the hippocampus, cortex and colon, as well as intestinal metabolites were evaluated. In vitro experiment, the polarization of microglia was assessed in an LPS+corticosterone (LPS&CORT) induced cellular stress model followed by intervention with *R.i.*-conditioned medium

The technical route for the present study is shown in Fig. 1.

### Clinical cohort study

Female adolescents (aged 11–17 years) firstly diagnosed with depression according to the Diagnostic and Statistical Manual of Mental Disorders, Fifth Edition (DSM-V) were recruited from Nanjing Brain Hospital affiliated with Nanjing Medical University (Jiangsu, China) between September 2020 and May 2021. Depression severity was assessed using the Revised Child Anxiety and Depression Scale-25 (RCADS-25) [24]. Participants with a parental history of bipolar disorder, schizophrenia, or other psychiatric disorders (*n* = 25) were excluded based on DSM-V criteria. Additional exclusion criteria included chronic systemic diseases (e.g., diabetes, cardiovascular or thyroid diseases, cancer), substance abuse, and current or recent bacterial, fungal, or viral infections. Healthy controls (HC, *n* = 20) were recruited matched by gender and other demographic characteristics. Baseline demographic and mental health information was collected using a standardized pediatric mental health questionnaire. The relevant demographic characteristics of the participants are presented in the Supplementary Materials (Table S1-2). None of the participants exhibited major psychiatric comorbidities.All were medication-naïve, with no use of antibiotics, anti-inflammatory agents, prebiotics, or antidepressants within the preceding two months. Fecal samples were collected from all participants. The study protocol was reviewed and approved by the Ethics Committee of Nanjing Medical University (Approval No.: 2020-KY 19801). Written informed consent was obtained from all participants prior to enrollment.

### 16 S rRNA gene sequencing

Total DNA was extracted from human fecal samples for 16S rRNA gene sequencing analysis. The V3-V4 region of the 16S rRNA gene was amplified using the primers 341F (5’-CCTACGGGNGGCWGCAG-3’) and 805R (5’-GACTACHVGGGTATCTAATCC-3’) with the KAPA HiFi HotStart PCR Kit (KAPA Biosystems, USA). The amplicon library was evaluated, and sequencing was performed on the Illumina NovaSeq PE250 platform. The raw sequencing reads were processed using QIIME 2 (version 2020.11). Alpha and beta diversity analyses were visualized using R packages. Taxonomic annotation of amplicon sequence variant (ASV) features was performed using the SILVA NT-16 S database (Release 138, https://www.arb-silva.de/documentation/release138/). Based on our prior 16 S rRNA gene sequencing data [13], we conducted the microbial community analysis for this study.

### Animal experiments

Female C57BL/6J mice (3 weeks old) were obtained from the National Rodent Laboratory Animal Resource Center (Shanghai, China) and housed under specific pathogen-free (SPF) conditions with controlled room temperature (21–23 °C), humidity (50–60%), and a 12 h/12 h light/dark cycle. All mice had ad libitum access to food and water and were acclimatized for one week prior to any experimental procedures. The chronic restraint stress (CRS) mice were established according to previously published protocols [25]. Briefly, mice were placed in well-ventilated 50 mL conical tubes that closely fitted their body size for 3–4 h daily for 28 consecutive days. After that, the behavioral tests were conducted. All animal experiments were approved by the Institutional Animal Care and Use Committee of Nanjing Medical University (Approval No.: IACUC-2412010-2).

GLPG0974 (HY-12940, MCE), a free fatty acid receptor 2 (FFAR2/GPR43) antagonist, was prepared as a 5 mg/mL working solution and administered intraperitoneally to mice at a dose of 100 µL every three days, for a total of 10 injections throughout the modeling period. The working solution was prepared as follows: 30 µL of a 50 mg/mL clear DMSO (ST038, Beyotime) stock solution was added to 250 µL of PEG300 (Y268724, Beyotime) and thoroughly mixed. Then, 720 µL of normal saline (ST341, Beyotime) was added to bring the final volume to 1 mL. Treg depletion in mice was achieved by intraperitoneal injections of anti-CD25 antibody (clone PC61, Bio X Cell, BE0012; 300 µg per dose administered on days − 1, 7, and 14 of CRS). Control mice received the IgG1 isotype (Bio X Cell, BE0088).

### Behavioral testing

Depression-like behaviors were assessed by the tail suspension test (TST) and forced swim test (FST), while anxiety-like behaviors were evaluated by the open field test (OFT) and elevated plus maze (EPM). The tests were scheduled sequentially to minimize carry-over effects, with the following order: OFT, EPM, TST, and FST. Detailed protocols and additional information are provided in the Supplementary Materials. All analyses were conducted in a blinded manner to ensure unbiased results.

### Tissue sample preparation

Mice were anesthetized with isoflurane (R510-22-10, RWD), and the absence of consciousness and pain reflex was confirmed. Intracardiac perfusion was performed with normal saline (ST341, Beyotime) until the liver became pale. The mice were then placed in a prone position. The skin along the midline of the skull was incised with scissors, fully exposing the cranium. With the ventral side facing up, the brain was carefully bisected along the longitudinal fissure into the left and right hemispheres. One hemisphere was placed in 4% paraformaldehyde (PFA) for fixation. The other hemisphere was transferred to a culture dish containing pre-cooled normal saline. The prefrontal cortex, located at the anterior part of the brain caudal to the olfactory bulbs, was carefully dissected away from the surrounding tissue using a scalpel. The hippocampus was identified as a curved, “C”-shaped structure on the medial aspect of the hemisphere. By gently separating along the interface between the cortex and the white matter and removing the overlying cortex, the hippocampus was isolated [26]. The dissected tissues were immediately stored at -80 °C.

### Bacterial strain

*R.i.* (DSMZ 14610) was resuscitated and cultured in reinforced clostridial medium (RCM). The components of RCM medium are as follows: 10.0 g/l peptone, 10.0 g/l beef extract powder, 3.0 g/l yeast extract powder, 5.0 g/l glucose, 1.0 g/l soluble starch, 5.0 g/l sodium chloride, 3.0 g/l sodium acetate, 0.5 g/l L-cysteine hydrochloride, and 0.5 g/l agar. The pH was adjusted to 6.8 ± 0.1 at 25 °C. The liquid medium was pre-purged with nitrogen for 2 h to ensure strict anaerobiosis, followed by incubation at 37 °C under anaerobic conditions for 48–72 h. The bacterial suspension for animal experiments was prepared as follows: *R.i.* cultures in the late exponential growth phase were harvested, resuspended in PBS, and administered to mice via oral gavage at a daily dose of 10⁹ colony-forming units (CFU) [27].

### Cell experiments

#### Stress and inflammatory cell model

BV2 murine microglial cells (Mingzhou Bio, China) were maintained in Dulbecco’s Modified Eagle Medium (DMEM, high glucose, Gibco, 11965092) supplemented with 10% fetal bovine serum (FBS, TransGen Biotech) and 1% penicillin/streptomycin (Hyclone) at 37 °C in a humidified incubator with 5% CO₂. For experiments, BV2 cells were seeded at a density of 5 × 10⁴ cells per well in 6-well plates (LABSELECT). Cells were then assigned to five groups: the control (CTR) group and four LPS+corticosterone (CORT) co-treatment groups with LPS concentration gradients (0.01, 0.1, 1, and 10 µg/mL). For the co-treatment groups, cells were exposed to LPS (at the designated concentration for each group) together with 0.1 µg/mL CORT for 24 h to establish the stress and inflammatory model.

#### Bacterial supernatant intervention

Supernatant Preparation: *R.i.* (Mingzhou Bio, China) was cultured for at least three generations. The bacterial culture was centrifuged (9000 rpm, 15 min), and the supernatant was filter-sterilized through a 0.22 μm membrane and stored at -80 °C. Heat Inactivation: Aliquots of the supernatant were sealed, heated at 95 °C for 5 min, and stored at -80 °C. Intervention Setup: BV2 cells were divided into six groups: Control (CTR) + reinforced clostridial medium (RCM), CTR + *R.i.* supernatant, LPS&CORT + RCM, LPS&CORT + *R.i.* supernatant, LPS&CORT + heat-inactivated *R.i.* supernatant, LPS&CORT + GLPG0974 + *R.i.* supernatant. After 2 h of seeding, cells were treated with 20% (v/v) of the respective supernatant or control medium, with or without 100 nM GLPG0974. After 6 h, the medium was replaced with DMEM containing 1 µg/mL LPS and 0.1 µg/mL CORT (except for control groups) for 24 h.

#### Quantitative analysis of short-chain fatty acids

Chromatography-grade methyl tert-butyl ether (MTBE) and a mixed short-chain fatty acid (SCFA) standard containing acetic acid, propionic acid, butyric acid, and 2-methylbutyric acid were purchased from ANPEL Laboratory Technologies (Shanghai, China). Fecal samples weighing 20 mg were homogenized and sonicated with 800 µL PBS. After centrifugation, 200 µL of the supernatant was collected and acidified with 2 µL HCl. Then, 400 µL of cold MTBE was added, the mixture was vortexed and centrifuged, the supernatant was transferred to a 1.5 mL brown glass vial for analysis. The mixed standard solution was serially diluted with MTBE to achieve concentration ranges from 0.1 to 10 µg mL⁻¹. Calibration curves for each SCFA were constructed by plotting the peak area against the corresponding analyte concentration. Analysis was performed using a Shimadzu QP2020 GC-MS system (Kyoto, Japan). Chromatographic separation was achieved using an Agilent DB-FATWAX MS UI capillary column (30 m × 0.25 mm × 0.25 μm; California, USA). The temperature program was set as follows: initial temperature of 50 °C held for 1 min, increased to 170 °C at a rate of 15 °C min⁻¹, and held for 3 min. The injection volume was 1 µL with a split ratio of 5:1. Helium was used as the carrier gas at a constant flow rate of 1.5 mL min⁻¹. Detection was performed in selected ion monitoring (SIM) mode. The temperatures of the GC-MS transfer line, ion source, and quadrupole were maintained at 280 °C, 250 °C, and 150 °C, respectively.

### Real-Time PCR (QPCR)

Total RNA was extracted from hippocampal tissues using TRIzol reagent (Invitrogen, Waltham, MA, USA) following the manufacturer’s protocol. RNA concentration and purity were assessed using a NanoDrop 2000 spectrophotometer (Thermo Fisher, USA) by measuring OD260/OD280 ratios. Complementary DNA (cDNA) was synthesized using the PrimeScript™ RT Master Mix (Takara, Japan) according to the manufacturer’s instructions. Quantitative PCR (qPCR) was performed using SYBR Green Supermix (Yeasen Biotech, China) on a Roche LightCycler 480 system (Switzerland). Relative mRNA expression levels were calculated using the 2^^−ΔΔCt^ method. The procedure is detailed as follows: First, the ΔCt for each sample is calculated by subtracting the mean Ct value of the endogenous reference gene from the Ct value of the target gene. Next, ΔΔCt is derived by subtracting the mean ΔCt of the control group from the ΔCt of each group. Finally, the fold change in gene expression is calculated as 2^^−ΔΔCt^. Primer sequences for all genes analyzed are provided in the Supplementary Materials (Table S3).

### Flow cytometry

#### Blood sample processing

Fresh blood was collected from the ocular venous plexus of experimental mice. Red blood cell lysis was performed using ACK buffer (9101, absin). The supernatant was discarded, and the cell pellet was resuspended in a defined volume of buffer. The cell suspension was transferred to staining tubes and incubated with the following antibody cocktail: CD3ε-FITC (11-0031-85, eBioscience, 10:90), CD4-BUV650 (100469, biolegend, 10:90), and CD25-BV421 (62-0251-82, eBioscience, 8:92), then 1 µL FVD-eFluor™ 780 (65-0865-14, eBioscience) was added, and the mixture was incubated in the dark. Fixation buffer (00-5523-56, 00-5123-43 Invitrogen, Thermo Fisher Scientific) was added at a 1:3 ratio, followed by an additional 30-minute incubation. Cells were washed with PBS and permeabilization buffer (00-8333-56, Invitrogen, Thermo Fisher Scientific), followed by centrifugation. Finally, the pellet was resuspended in permeabilization buffer supplemented with anti-Foxp3 antibody (17-5773-82, Thermo Fisher Scientific), and the mixture was incubated prior to sample acquisition. The final detection concentrations for CD3ε-FITC, CD4-BUV650, CD25-BV421, and the anti-Foxp3 antibody were 0.5 µg/100 µL, 0.25 µg/100 µL, 0.125 µg/100 µL, and 1 µg/100 µL, respectively.

#### Colon sample processing

Colon tissues were processed according to the manufacturer’s instructions of the Gentle Enzyme Dissociation Kit for Mouse Intestinal Tissue (RWD Life Science, DHIE-5007). The specific procedures were as follows: Colon tissues were isolated from mice. After removing contaminants and fecal contents, the tissues were washed thoroughly and cut into 2–4 mm fragments. The fragments were vortex-washed with PBS and subsequently washed with pre-warmed wash buffer under constant agitation. The tissue suspension was then passed through a 100 μm cell strainer. The retained tissue fragments were transferred into a digestion tube containing freshly prepared enzyme cocktail and processed using a single-cell suspension preparation instrument according to the manufacturer’s protocol. Following enzymatic digestion, the cell suspension was filtered, centrifuged, and the supernatant was discarded. Finally, the cell pellet was resuspended in appropriate buffer. For staining, the following reagents were sequentially added: CD45-Percp-Cy5.5 (45-0451-82, eBioscience), CD3ε-FITC (11-0031-85, eBioscience), CD4-BV650 (100469, biolegend), CD25-BV421 (62-0251-82, eBioscience), and FVD-eFluor™ 780 (65-0865-14, eBioscience), then the mixture was incubated in the dark. The fixation buffer (Invitrogen, Thermo Fisher Scientific) was added at a 1:3 ratio, followed by an additional 30-minute incubation. Cells were washed with PBS and permeabilization buffer(Invitrogen, Thermo Fisher Scientific), followed by centrifugation. Finally, the pellet was resuspended in permeabilization buffer supplemented with anti-Foxp3 antibody (17-5773-82, Thermo Fisher Scientific), and the mixture was incubated prior to sample acquisition. The final detection concentrations for CD45-Percp-Cy5.5, CD3ε-FITC, CD4-BUV650, CD25-BV421 and the anti-Foxp3 antibody were 0.125 µg/100 µL,0.5 µg/100 µL, 0.25 µg/100 µL, 0.125 µg/100 µL, and 1 µg/100 µL, respectively.

### Immunofluorescence staining

The mouse brain tissue, following perfusion with saline, was fixed using a 4% paraformaldehyde solution. After dehydration, tissues were embedded in the Optimal Cutting Temperature (O.C.T.) compound, and hippocampal coronal brain Sects.  (25–30 μm thickness) were prepared. Sections were blocked with 5% goat serum and permeabilized with 0.3% Triton X-100 (Sigma-Aldrich, St. Louis, MO, USA). Primary antibodies were incubated overnight at 4 °C, including anti-DCX (4604 S; Cell Signaling Technology, MA, USA), anti-IBA1 (019-19741; WAKO, Tokyo, Japan), NeuN Monoclonal antibody (66836-1-Ig, proteintech). Afterward, sections were incubated with secondary antibodies (Alexa Fluro^®^ 594 goat anti-rabbit IgG, Invitrogen; Multi-rAb™ CoraLite^®^ Plus 594-Goat Anti-Mouse Recombinant Secondary Antibody (H + L), proteintech), then washed, and mounted using DAPI mounting medium (ab104139, Abcam). Microscopic images were acquired using a Zeiss LSM900 scanning laser confocal microscope (Carl Zeiss, Oberkochen, Germany). For microglia analysis, 3D reconstruction of microglial cells was performed using a confocal laser scanning microscope with Z-axis series captured at 1.0 μm intervals. Morphological characteristics of microglia were recorded and analyzed using IMARIS software (version 9.0.1, Bitplane, Switzerland). DCX^+^ cells were manually counted across the entire fluorescence microscopy field of view. The counts were subsequently normalized to the area of the field and expressed as the number of DCX-positive cells per unit area. For NeuN-positive neurons, cell counting was employed to quantify neurons at the transition zone from the dentate gyrus (DG) to the CA3 region of the hippocampus.

### Statistical analysis

Statistical analyses were conducted using GraphPad Prism (Version 8.0). Data are presented as the mean ± standard deviation (SD) or standard error of the mean (SEM). For comparison across multiple groups, one-way or two-way analysis of variance (ANOVA) was performed with appropriate between- and within-group factors, followed by Tukey’s post hoc tests for pairwise comparisons. For comparisons between two groups, if the data were normally distributed and demonstrated homogeneity of variance, an unpaired t-test was employed. Otherwise, the non-parametric Mann-Whitney U test was applied. Statistical significance was defined as *p* < 0.05.

## Results

### Alterations of the intestinal microbiota in the unmedicated‑depressive adolescents

**Fig. 2:**
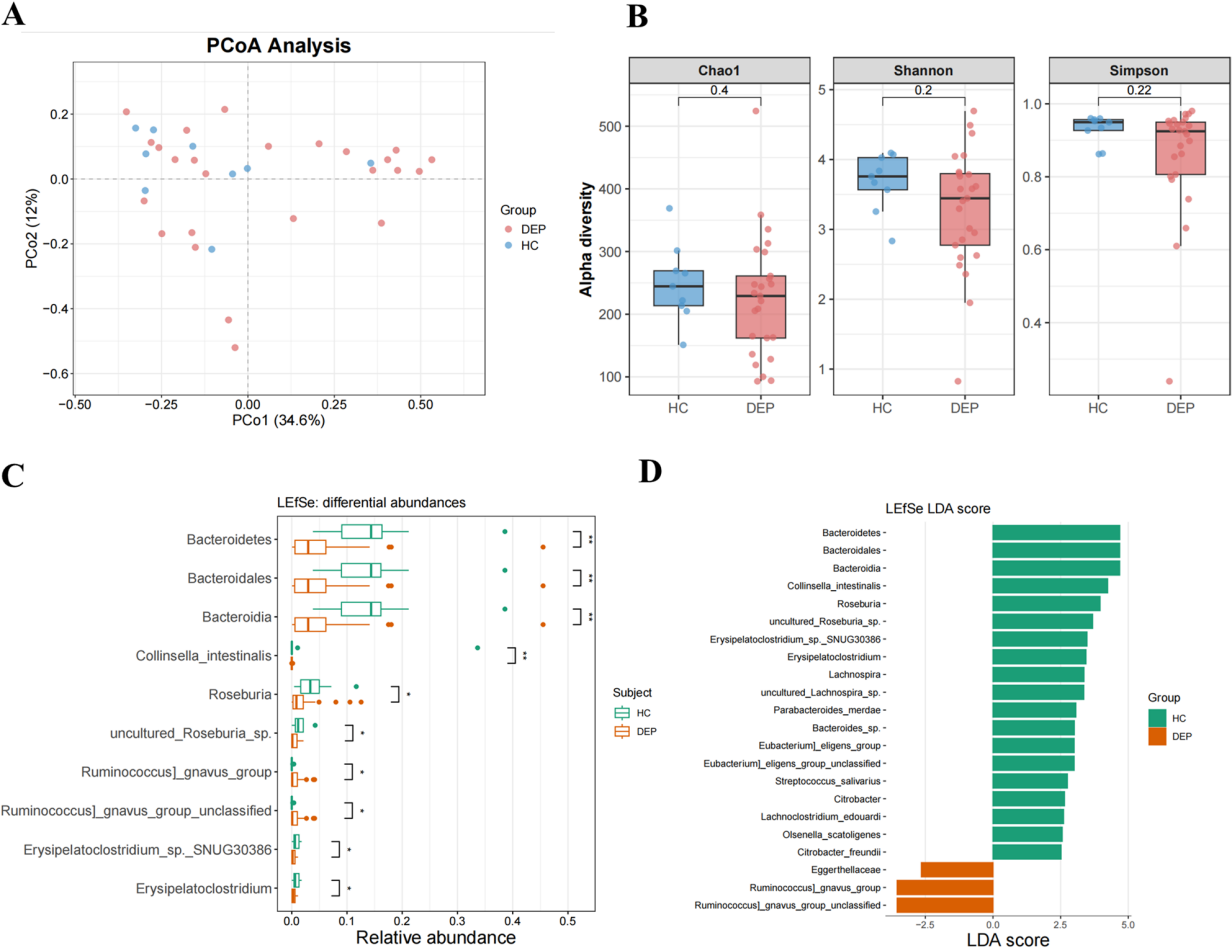
Changes of gut microbial composition in control and DEP group. **A **β-diversity index analysis based on Bray-Curtis dissimilarities (PCoA). **B** Chao1, Shannon and Simpson indices of α diversity based on numbers of features. **C** Analysis of Differentially Abundant Taxonomic Units in the Microbiota. **D** Linear discriminant analysis effect size (LEfSe) analysis identified a distinctive gut microbiota composition characteristic of the DEP group. Data are presented as mean ± SD. p values were obtained by a Wilcoxon test. *p < 0.05, **p < 0. 01

Female unmedicated depressive adolescents (DEP) and matched healthy controls (HC) were enrolled in this study, with diagnoses confirmed by two attending psychiatrists or one senior clinical psychiatrist. To elucidate the potential association between gut microbiota and depression, we analyzed the composition and functional characteristics of the gut microbiome in HC and DEP. Principal coordinates analysis (PCoA) revealed significant differences in microbial community composition between the HC and DEP groups (Fig. 2A). The Shannon index, Simpson index and Chao1 index showed a downward tendency though with no statistical significance (Fig. 2B). The Shannon index reflects the integrated characteristics of species richness and evenness within a microbial community; the Simpson index focuses on measuring the concentration of dominant species within the community and is more sensitive to changes in these dominant species; the Chao1 index is primarily used to estimate the total species richness in a community. Synthesizing these results, it can be inferred that there is no pronounced alteration in the alpha-diversity of the gut microbiota between patients with depression and the healthy control group. To investigate the association between altered gut microbiota and depression, we employed the Linear Discriminant Analysis Effect Size (LEfSe) method to analyze differences in the abundance of taxonomic units in the gut microbiota between HC and patients. The results revealed that the relative abundance of *Roseburia* genus was significantly higher in HC compared to that in DEP patients (Fig. 2C). The corresponding LEfSe LDA score plot (Fig. 2D) indicated *Roseburia* was a signature taxonomic unit enriched in HC, while its abundance was reduced in DEP patients.

### Fecal butyrate levels are specifically reduced and negatively correlated with depression severity in adolescent females with MDD

**Fig. 3:**
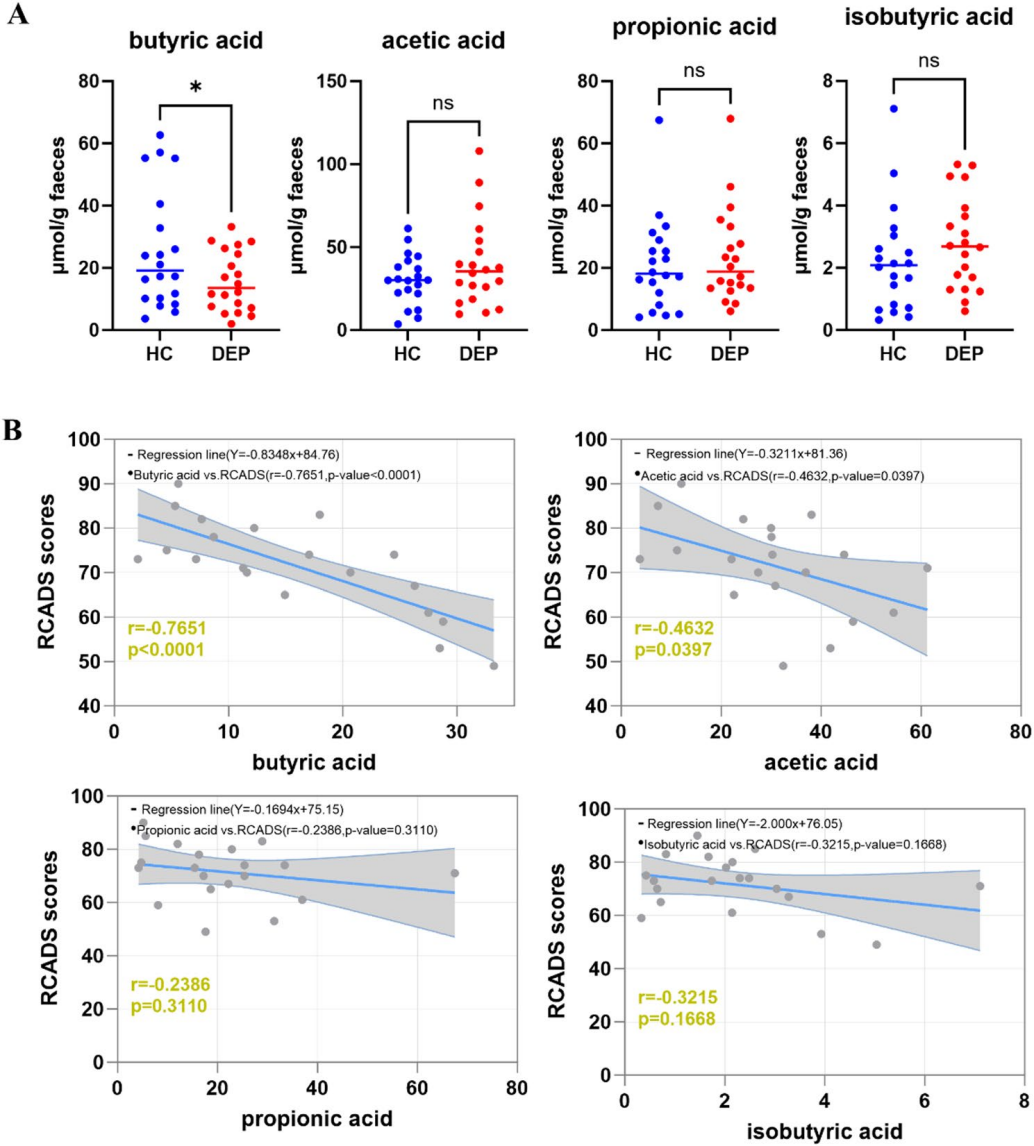
Association between SCFAs and adolescent depression. **A** Concentrations of SCFAs in the depression (DEP) group and healthy control (HC) group. **B** Correlation between RCADS-25 scores and faeces SCFAs levels. All data are presented as mean ± SD. *p < 0.05, ns, no significance

Given the aforementioned distinct gut microbial compositions between DEP and HC, specifically, the reduced abundance of *R.i.* at the genus level in the DEP group relative to the HC group, then we detected faecal short-chain fatty acid (SCFA) levels, since a close association between *R.i.* and SCFAs, particularly butyrate. As expected, fecal butyrate levels were significantly decreased in depressed adolescents detected by GC-MS, while the concentrations of acetate, propionate, and isobutyrate exhibited no obvious changes (Fig. 3A). These alterations suggest that the decreased butyrate levels in depressed adolescents may be associated with the *R.i.* depletion. On the basis of this assumption, the correlation between SCFA levels and RCADS depression scores were evaluated. Notably, butyrate exhibited a significant negative correlation with RCADS scores (*r* = − 0.7651, *p* < 0.0001), and the acetate displayed a weaker but statistically significant negative correlation (*r* = − 0.4632, *p* = 0.0397), whereas propionate (*r* = − 0.2386, *p* = 0.3110) and isobutyrate (*r* = − 0.3215, *p* = 0.1668) showed no significant correlations (Fig. 3B).

### *R.i.* increases butyrate production and exerts antidepressant effects in a GPR43-dependent manner

**Fig. 4:**
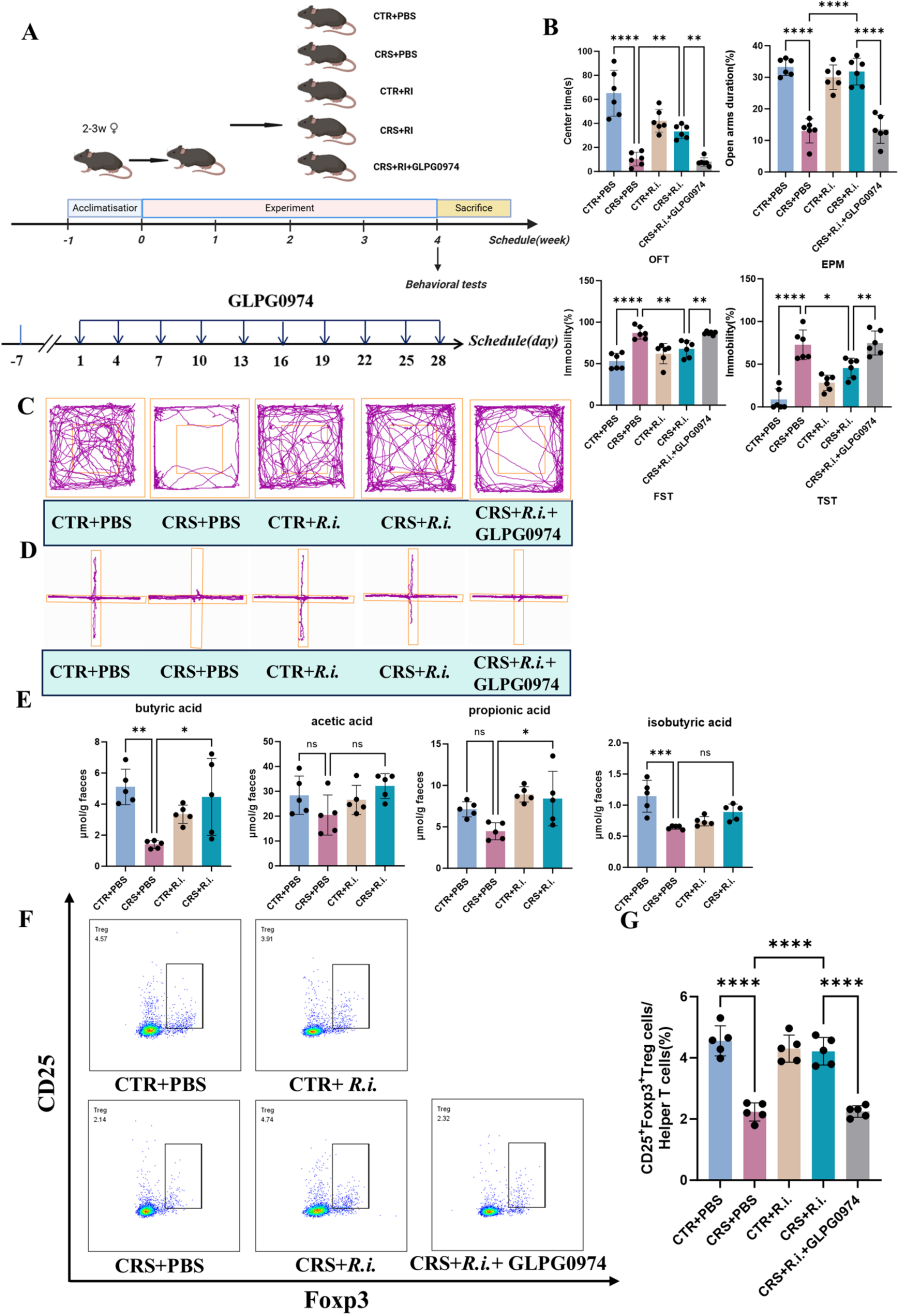
Effects of *R.i.* transplantation on chronic restraint stress (CRS)-induced behavioral changes and Treg cell responses. **A** Schematic diagram of the animal experimental timeline and flow chart. **B** Behavioral performance across experimental groups: center time (s) in the open field test (OFT), percentage of time spent in open arms of the elevated plus maze (EPM), and immobility rate (%) in both the forced swim test (FST) and tail suspension test (TST). *n* = 6 mice per group. **C** Representative movement trajectories in the OFT. **D** Representative movement trajectories in the EPM. **E** Relative concentrations of fecal SCFAs in each group of mice. *n* = 5 mice per group. **F** Representative flow cytometry plots of Treg cells in the blood of mice. **G** Quantitative analysis of the relative proportion of Treg cells in the blood of mice. *n* = 5 mice per group. All data are presented as mean ± SD. *p < 0.05, **p < 0.01, ***p < 0.001, ****p < 0.0001. ns, not significant; CTR, Control

To assertain the causal relationship bewteen *R.i.* abundance and depression, the *R.i.* was transplatated to the chronic restraint stress (CRS) depressed adolescent mice. Behavioral results showed that compared to the CRS group, mice in the CRS + *R.i.*group exhibited significantly prolonged center time in the open field test (OFT) and elevated plus maze (EPM), along with markedly reduced immobility time in the forced swim test (FST) and tail suspension test (TST), indicating that *R.i.* ameliorated depression- and anxiety-like changes. Then, the mice transplanted with *R.i.* and concurrently treated with GLPG0974 (Fig. 4A), the antagonist of the butyrate receptor FFAR2 (G-protein coupled receptor 43, GPR43). As shown in Fig. 4B-D, the salutary effects of *R.i.* in CRS mice were dramatically weakened, manifesting as shortened time in the center of the OFT and EPM, and prolonged immobility time in the FST and TST, underscoring the key roles of FFAR2 in *R.i.* mediated antidepressant effects.

GC-MS analysis of fecal SCFAs revealed a significant decline of butyrate levels in CRS mice, an effect that was obviously ameliorated by the *R.i.* transplantation. Meanwhile, the propionate levels in CRS mice displayed a soft decline, and this effect also markedly reversed after *R.i.* transplantation (Fig. 4E). These results suggest that the alleviation of depression- and anxiety-like behaviors by *R.i.* may partially be associated with butyrate production.

### *R.i.* transplatation reverses the Treg cell decrease and inflammation in CRS mice

Since butyrate is closely associated with inflammation and CD4^+^ T cell differentiation [28], then the potential effects of *R.i.* transplatation on Treg cell modulation was investigated. As shown in Fig. 4F-G, the Treg cells were substantially decreased in CRS mice, noteworthily, *R.i.* treatment markedly restored Treg cell counts in mouse blood, and this effects, was remarkablly impeded by the blokage of the FFAR2, underlying the pivotal roles of butyrate and FFAR2 in Treg cell regulation.

**Fig. 5:**
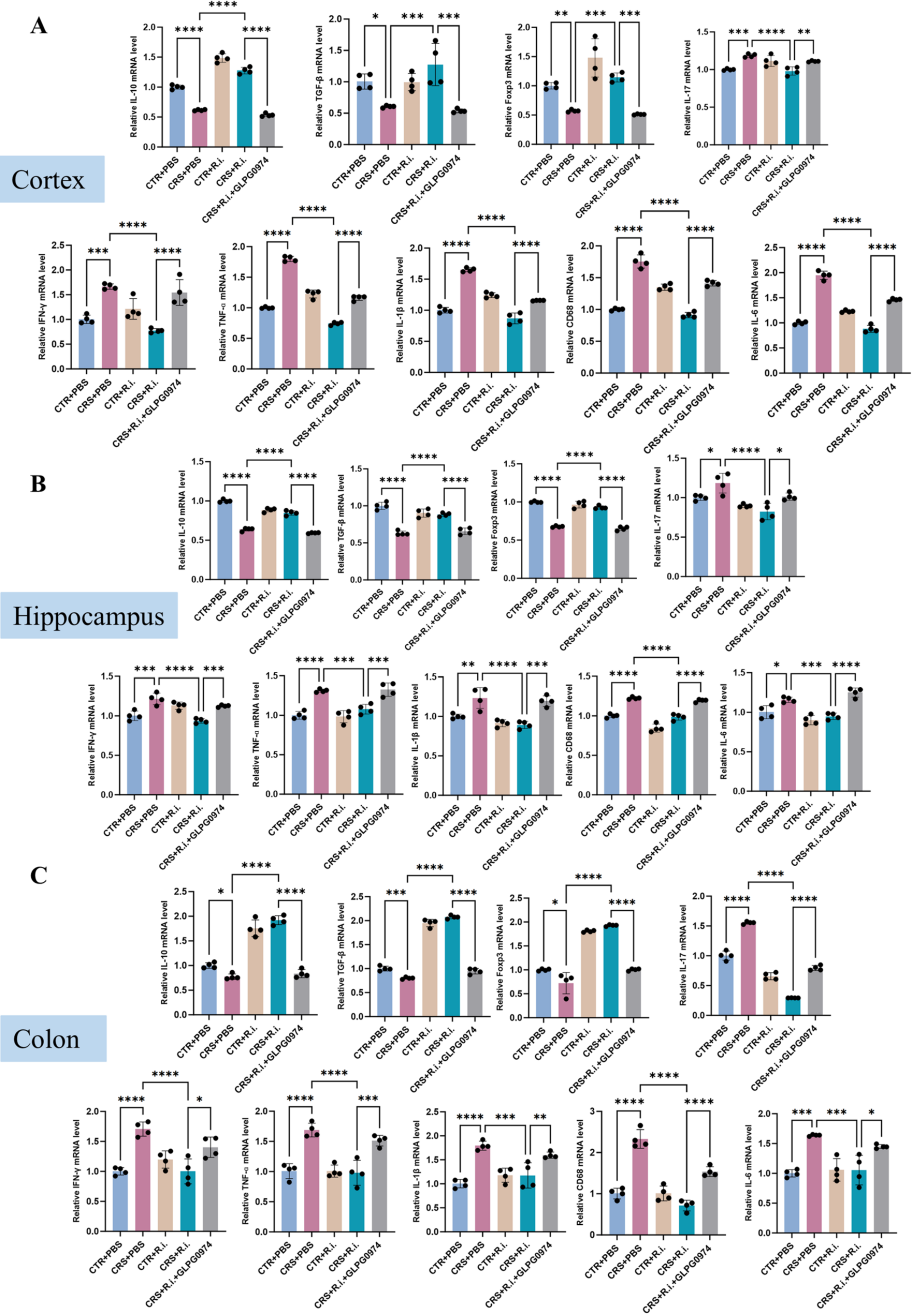
R.i. treatment improves inflammatory cytokine profiles in cortex, hippocampus, and colon of CRS mice. **A**-**C** Expression levels of pro-inflammatory cytokines, anti-inflammatory cytokines, and transcription factors in the cortex (**A**), hippocampus (**B**), and colon (**C**). *n* = 4 for each group. All data are presented as mean ± SD. *p < 0.05, **p < 0.01, ***p < 0.001, ****p < 0.0001

Having determined the effects of *R.i.* transplatation on expanding Treg cells and considering the crucial functions of Treg cells on suppressing Th17 cells and controlling inflammation, then we examined whether *R.i.* transplatation ameliorated CRS induced inflammation in the brain and colon. The hippocampus is highly susceptible to microglial activation and pro-inflammatory cytokines (e.g., IL-1β, IL-6, TNF-α), while the prefrontal cortex acts as a key hub for higher-order cognition and emotional regulation, and dysfunction in this region is directly linked to the core symptoms of depression [29, 30]. Therefore, to comprehensively elucidate how neuroinflammation drives depressive-like behaviors through functional dysregulation, we assessed the inflammatory status in these two brain regions. It showed that the anti-inflammatory cytokines (TGF-β and IL-10) were markedly decreased in the CRS mice, reciprocally, the pro-inflammatory cytokines (including IL-17, TNF-α, IFN-γ, IL-1β, IL-6) and cluster of differentiation 68 (CD68) were dramatically elevated. Besides, the Treg transcription factor Foxp3 was also reduced in the brain and colon, whereas *R.i.* treatment normalized the Foxp3 levels. Intriguingly, the anti-inflammatory effects of *R.i.* were dramatically attenuated by FFAR2 inhibition (Fig. 5A-C).

### *R.i.* transplatation reverses microglial activation and neurogenesis impairment in CRS mice

**Fig. 6:**
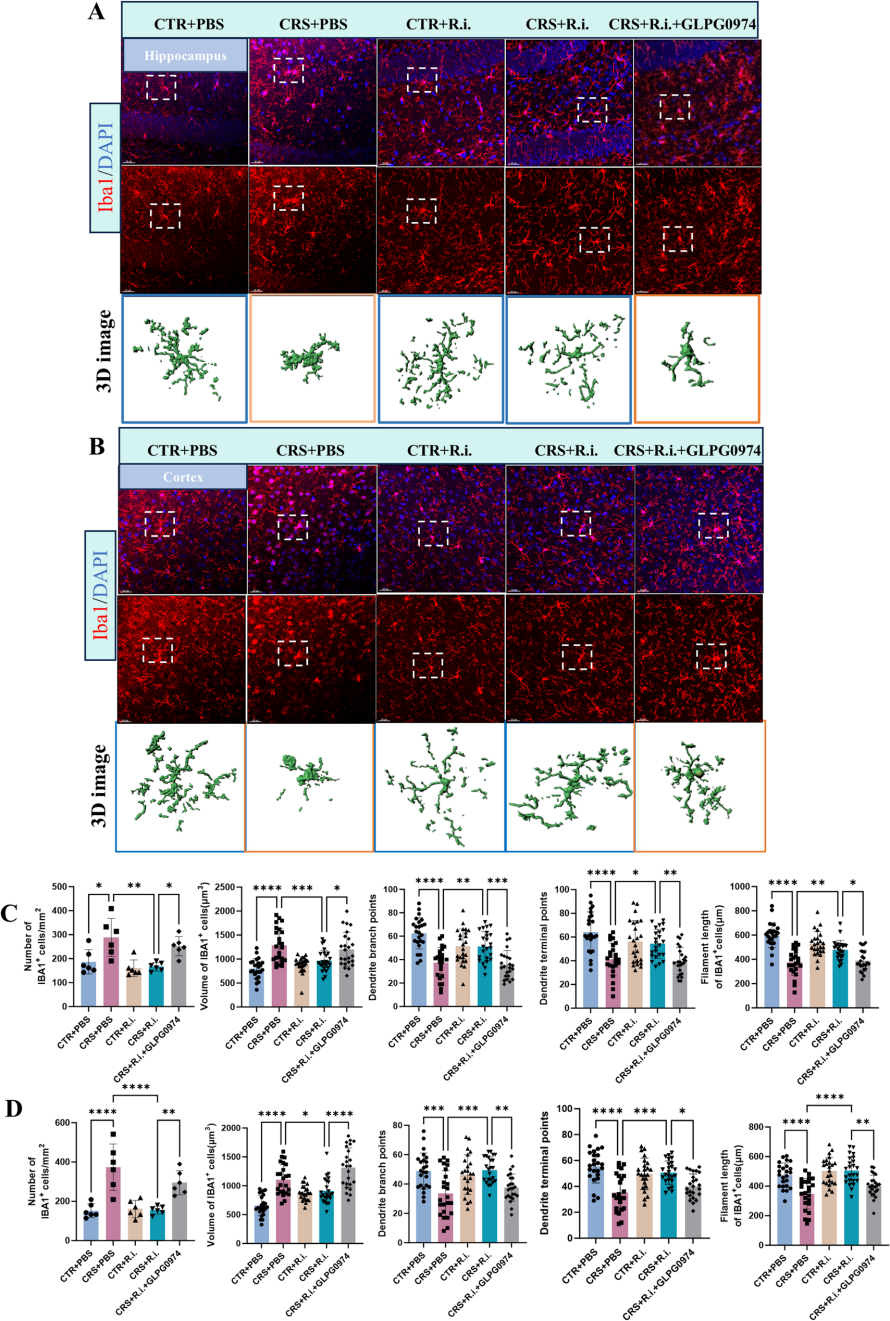
*R.i.* treatment exerts beneficial effects on CRS-induced microglial morphological changes. **A**-**B** Representative immunofluorescence images of microglia (IBA1⁺ cells) in the hippocampus and cortex, along with semi-automated 3D reconstructions of IBA1⁺ cells generated using IMARIS. Scale bar, 30 μm. *n* = 6 mice per group, 4 microglial cells were selected from immunofluorescent sections of each mouse for analysis. **C**-**D** Quantitative analysis of microglial morphology in the hippocampus and cortex, including cell number, cell volume, filament length, dendrite branch point number, and dendrite terminal point number of microglial cells. *n* = 6 mice per group, 4 microglial cells were selected from immunofluorescent sections of each mouse for analysis. All data are presented as mean ± SD. *p < 0.05, **p < 0.01, ***p < 0.001, ****p < 0.0001

**Fig. 7:**
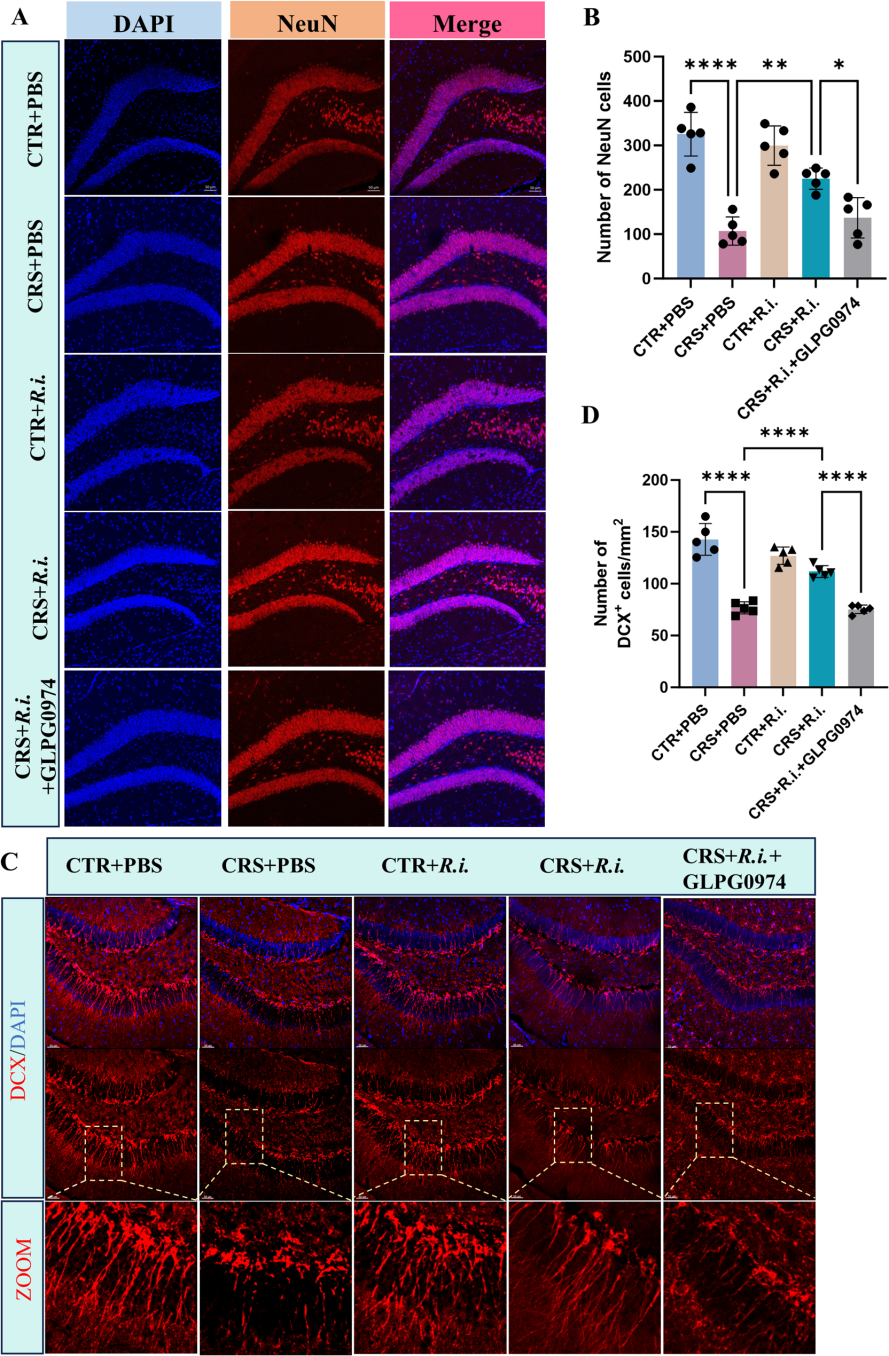
*R.i.* treatment attenuates the reduction in hippocampal neuronal cell count induced by CRS. **A** Representative immunofluorescence images of Neuronal Nuclei (NeuN) in the hippocampal region. Scale bar, 50 μm. **B** Quantitative analysis of NeuN cells. *n* = 5 mice per group. **C** Immunofluorescence micrographs depicting immature neurons (DCX⁺ cells) in the hippocampal region. Scale bar, 50 μm. **D** Quantitative analysis of DCX⁺ cells. *n* = 5 mice per group. All data are presented as mean ± SD. *p < 0.1, **p < 0.01, ****p < 0.0001

Given the obvious neuroinflammation induced by CRS and the anti-neuroinflammation of *R.i.* in mice aformentioned, we then sought to examine the potential effects on microglial morphology and neurogenesis. For microglia analysis, 3D reconstruction of microglial cells demonstrated that CRS elicited a pronounced microglial overactivation, characterized by increased microglial numbers, hypertrophic cell bodies, shortened processes, and reduced branching and endpoints. Intriguingly, *R.i.* transplantation notably reversed these deleteriorated outcomes, restoring microglial complexity, cell density, and soma size to levels comparable to the control group, while also increasing the dendritic length, branch points, and terminal points. As expected, the FFAR2 was participated in the *R.i.* mediated salutory effects, as evidenced by the ability of the GLPG0974 to ameliorate the *R.i.* mediated anti-microglial activation (Fig. 6A-D). Furthermore, the results demonstrated that CRS significantly impaired hippocampal neurogenesis and reduced neuronal numbers, as evidenced by markedly reduced expression levels of the immature migrating neuron marker doublecortin-positive (DCX^+^) neurons, and the mature neuron marker Neuronal Nuclei (NeuN). In contrast, *R.i.* treatment significantly restored the expression of all two markers, a protective effect that was ameliorated by GLPG0974 treatment (Fig. 7A-D). These findings indicate that *R.i.* transplantation reverses the CRS-induced aberrations in microglial morphology and cell population in the mouse brain, facilitates the restoration of neuronal cell populations, and thereby mitigates CRS-induced behavioral deficits. Importantly, all these beneficial effects were abrogated following FFAR2 blockage, underscoring the pivotal roles of butyrate receptor signaling in the favorable actions of *R.i.*.

### Depletion of Treg cells abolishes the antidepressant efficacy of *R.i.*

**Fig. 8:**
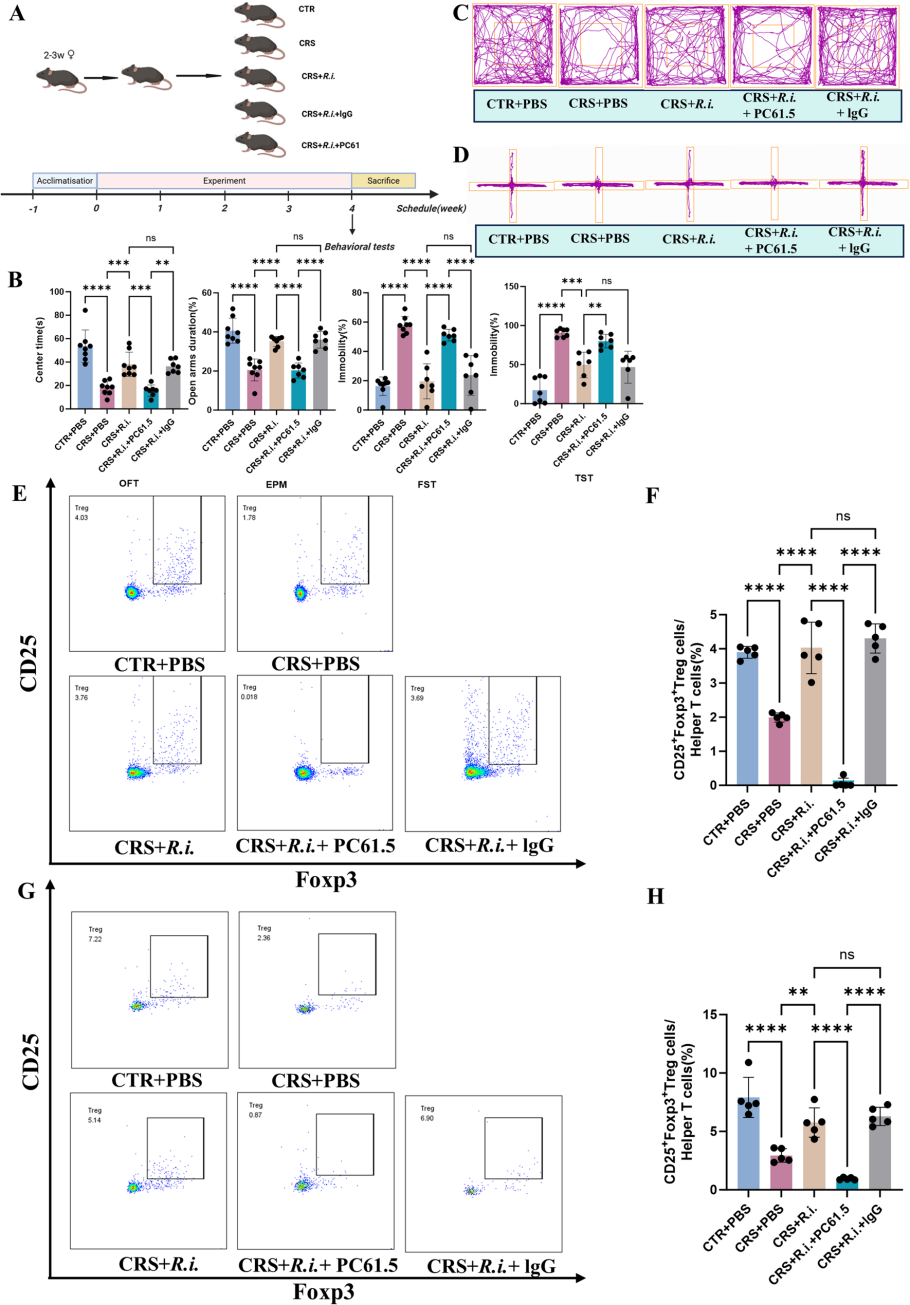
Treg cell depletion abolishes the beneficial effects of *R.i.* on depression-like behaviors in mice. **A** Schematic diagram of the experimental timeline. **B** Behavioral performance across groups: center time (s) in the open field test (OFT), percentage of time spent in open arms of the elevated plus maze (EPM), and immobility rate (%) in the forced swim test (FST) and tail suspension test (TST). *n* = 6–7 mice per group. **C** Representative movement trajectories in the OFT. **D** Representative movement trajectories in the EPM. **E** Representative flow cytometry plots of Treg cells in blood. **F** Quantitative analysis of Treg cell proportion in blood. *n* = 5 mice per group. **G** Representative flow cytometry plots of Treg cells in colon. **H** Quantitative analysis of Treg cell proportion in colon. *n* = 5 mice per group. All data are presented as mean ± SD. **p < 0.01, ***p < 0.001, ****p < 0.0001; ns, not significant

Given the expanding Treg cell levels in blood following *R.i.* transplantation in depressed mice, we sought to determine whether Treg cells are involved in the amelioration of depression- and anxiety-like behaviors by *R.i.* To this end, we established a CRS mouse model in which Treg cells were depleted using a CD25-neutralizing antibody (PC61.5) (Fig. 8A). Mice subjected to CRS were treated with *R.i.* while simultaneously receiving PC61.5 to deplete Treg cells, with an IgG antibody used as an isotype control. As shown in Figure S1B, the PC61.5 antibody effectively depleted over 95% of peripheral Treg cells. Behavioral results indicated that, compared to the CRS group, mice in the CRS + *R.i.* group exhibited significantly longer center time in OFT and EPM, as well as markedly reduced immobility time in FST and TST, indicating the beneficial effect of *R.i.* on depressive- and anxiety-like behaviors. In contrast, Treg depletion via PC61.5 abolished these improvements, as evidenced by significantly shortened center time in OFT and EPM and prolonged immobility time in FST and TST, and behavioral performance in the CRS + *R.i.* + IgG group was comparable to that in the CRS + *R.i.* group (Fig. 8B-D).

The flow cytometry analysis of Treg cells in blood and colonic tissue revealed a significant reduction in the CRS group compared to controls. *R.i.* intervention restored Treg cell levels, whereas the CRS + *R.i.* + PC61.5 group showed a sharp decrease of the Treg celss, confirming the efficient and specific Treg depletion by the CD25-neutralizing antibody. In parallel, the CRS + *R.i.* + IgG group exhibited Treg levels similar to those in the CRS + *R.i.* group (Fig. 8E-H).

**Fig. 9:**
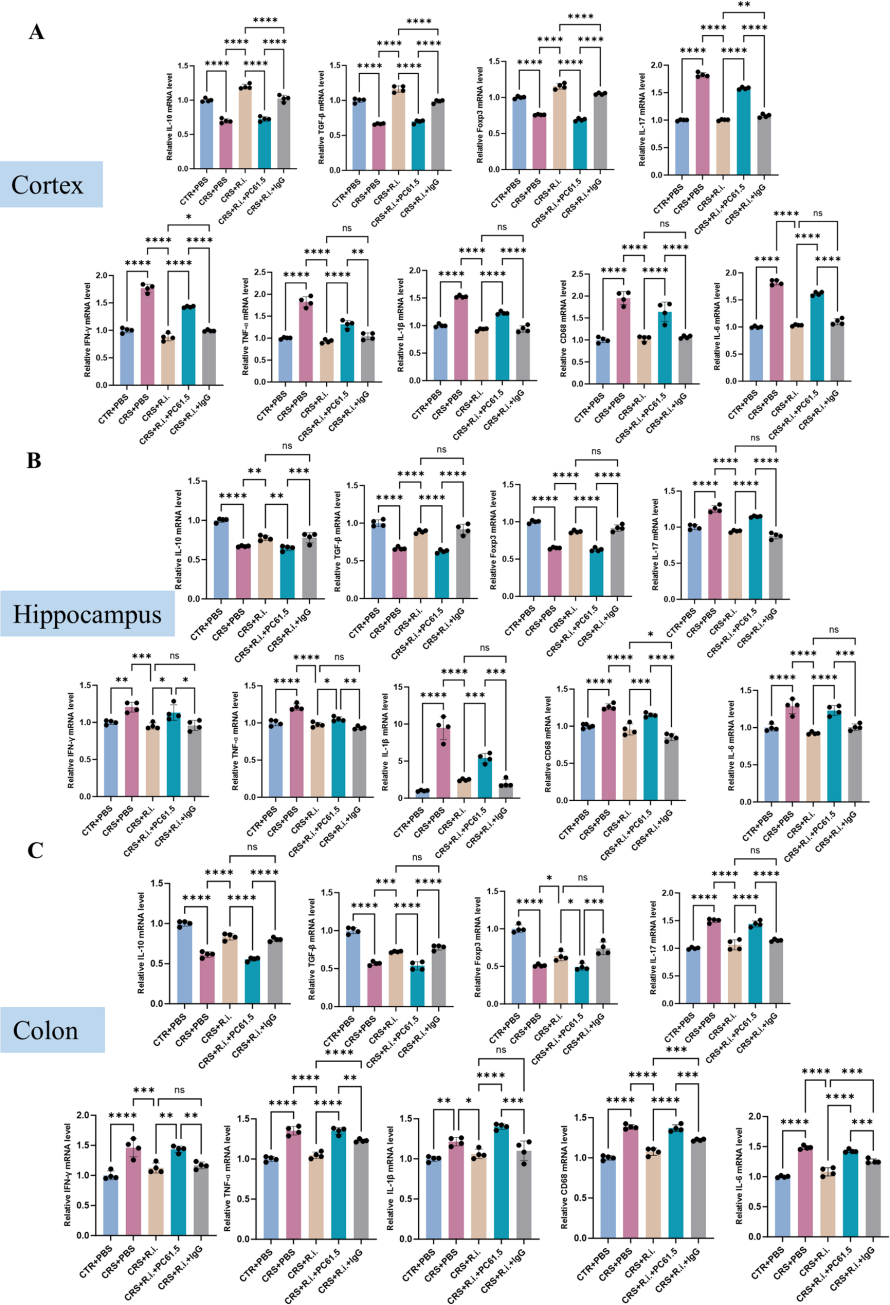
Depletion of Treg cells abolishes the anti-inflammatory effects of *R.i.* on the cortex, hippocampus, and colon of CRS mice. **A**-**C** Expression levels of pro-inflammatory cytokines, anti-inflammatory cytokines, and transcription factors in the cortex (**A**), hippocampus (**B**), and colon (**C**). *n* = 4 mice per group. All data are presented as mean ± SD. *p < 0.05, **p < 0.01, ***p < 0.001, ****p < 0.0001; ns, not significant

After the assessment of the efficacious Treg depletion, we examine the inflammatory effects in the cortex, hippocampus, and colon. Compared to the control mice, CRS mice exhibited significant decrease of the anti-inflammatory cytokines IL-10 and TGF-β, and reciprocally elevated the levels of the pro-inflammatory factors such as IL-17, TNF-α, IFN-γ, IL-6, and CD68, along with reduced Foxp3 expression. *R.i.* treatment obviously reversed these deteriorated effects, and Treg depletion substantially retarded the *R.i. mediated* beneficial effects. Inflammatory and transcriptional profiles in the CRS + *R.i.* + IgG group were comparable to those in the CRS + *R.i.* group (Fig. 9A-C).

**Fig. 10:**
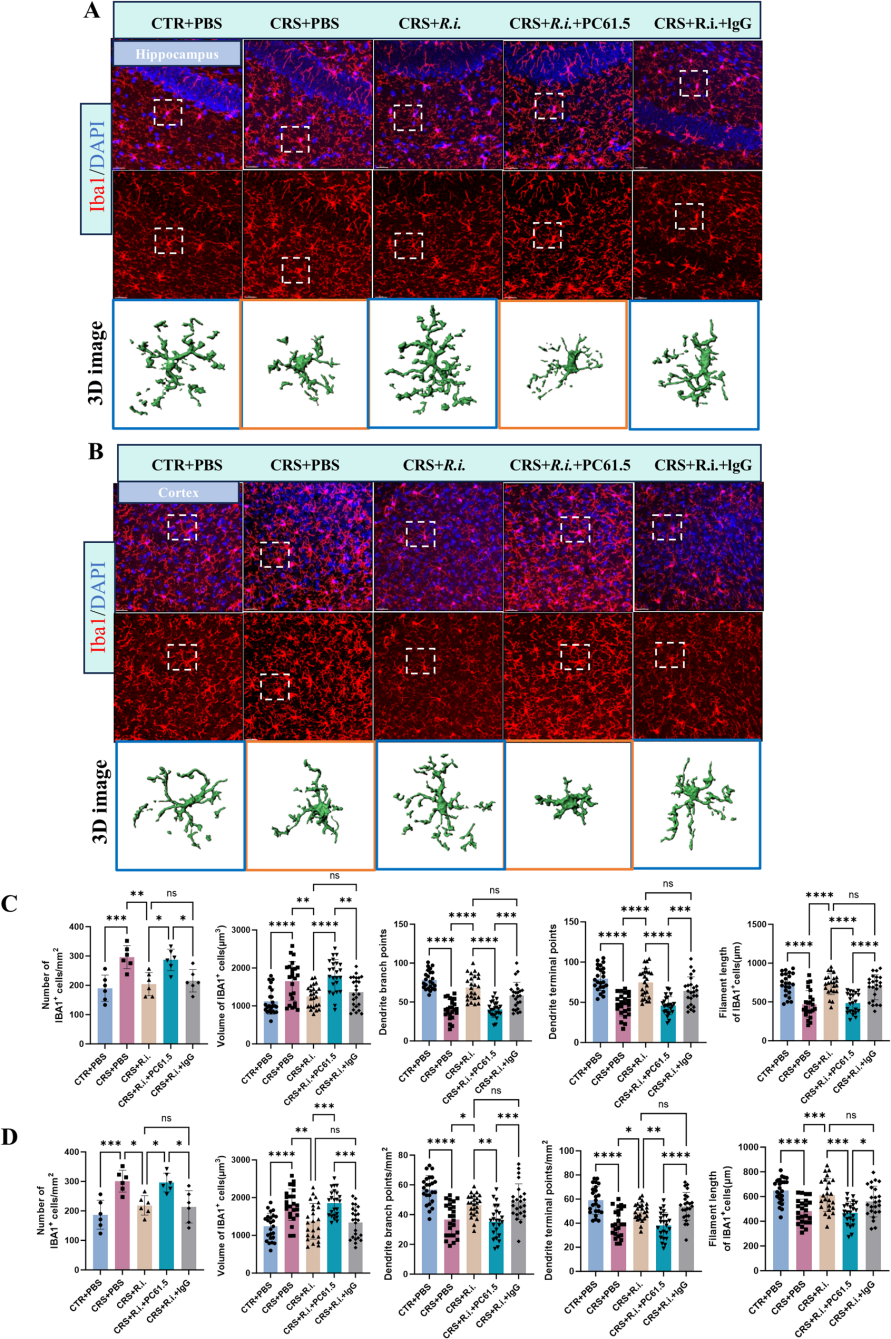
Depletion of Treg Cells abolishes the beneficial effects of *R.i.* on CRS-induced aberrant microglial morphology. **A**-**B** Representative immunofluorescence images of microglia (IBA1⁺ cells) in the hippocampus (**A**) and cortex (**B**), along with semi-automated 3D reconstructions of IBA1⁺ cells generated by IMARIS. Scale bar, 30 μm. **C**-**D** Quantitative analysis of microglial morphology in the hippocampus (**C**) and cortex (**D**). *n* = 6 mice per group, 4 microglial cells were selected from immunofluorescent sections for analysis in each mouse. All data are presented as mean ± SD. *p < 0.05, **p < 0.01, ***p < 0.001, ****p < 0.0001; ns, not significant

**Fig. 11:**
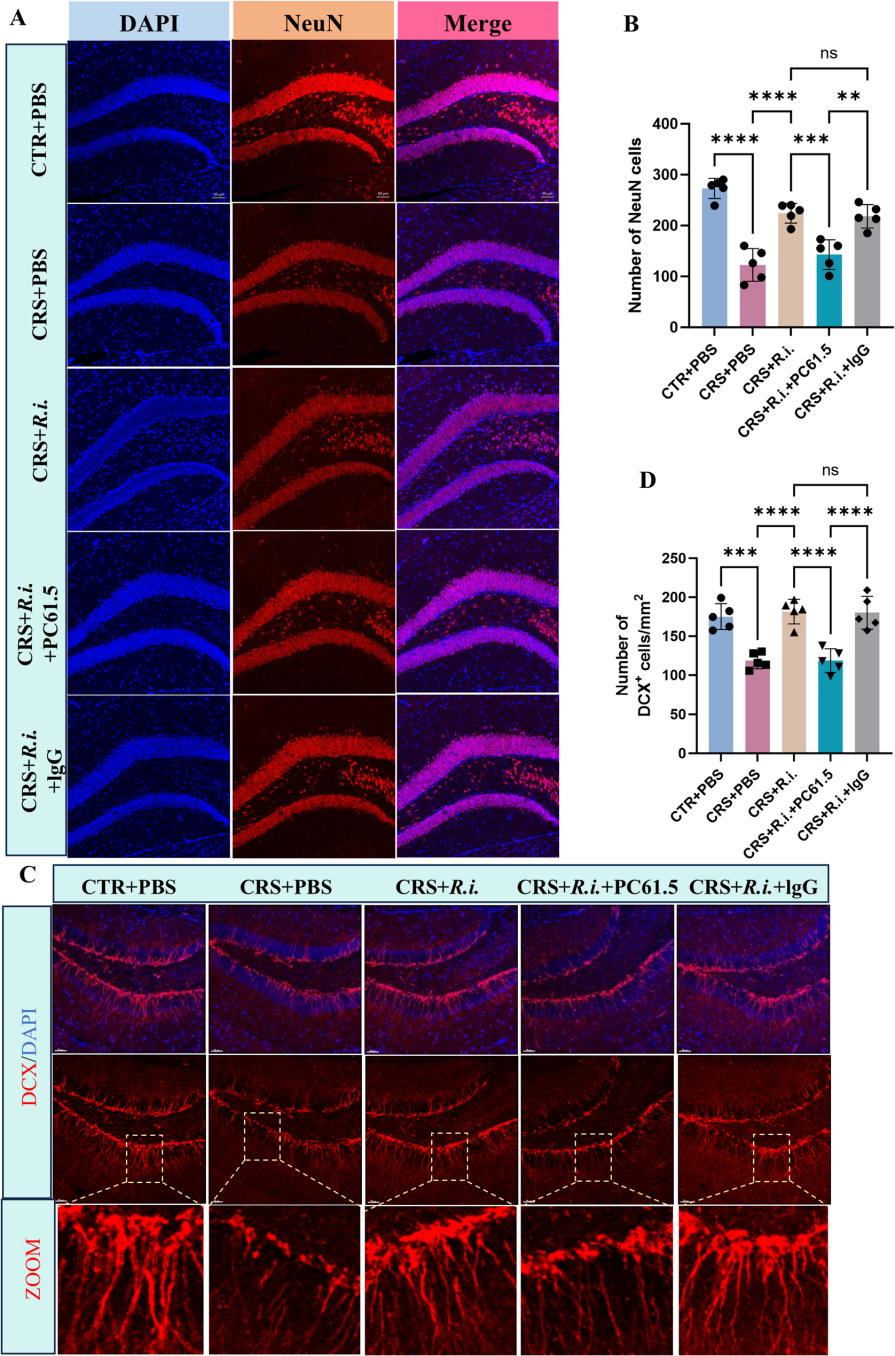
Depletion of Treg Cells abrogates the beneficial effects of *R.i.* against CRS-induced neuronal loss. **A** Representative immunofluorescence images of NeuN in the hippocampal region. Scale bar, 50 μm. **B** Quantitative analysis of NeuN cells. *n* = 5 mice per group. **C** Immunofluorescence micrographs depicting immature neurons (DCX⁺ cells) in the hippocampal region. Scale bar, 50 μm. **D** Quantitative analysis of DCX⁺ cells. *n* = 5 mice per group. All data are presented as mean ± SD. **p < 0.01, ***p < 0.001, ****p < 0.0001; ns, not significant

To further validate the role of Treg cells in *R.i.*-mediated mitigation of microglial activation and neuroinflammation, the microglial morphology and neurogenesis were examined. Quantitative analysis demonstrated that *R.i.* transplantation (CRS + *R.i.* group) significantly alleviated CRS-induced microglial abnormalities, restoring microglial branching complexity, cell density, and soma size to the level comparable to those in the control group, while simultaneously increasing dendritic length, branch points, and endpoints. In contrast, Treg depletion exhibited microglial hyperactivation similar to that of the CRS group, characterized by increased cell numbers, enlarged somata, shortened processes, and reduced branching complexity and endpoints (Fig. 10A-D). Furthermore, the pro-neurogenic effects mediated by *R.i.* were impaired after Treg cell depletion, as evidenced by significant reductions of DCX⁺ cells and the level of NeuN, a pattern similar to that observed in CRS mice (Fig. 11A-D).

These findings indicate that *R.i.* transplantation reverses the CRS-induced aberrations in microglial morphology, facilitates the restoration of neuronal numbers and enhances neurogenesis, a process in which the Treg cells play a pivotal role.

### *R.i.*-derived metabolites directly polarize microglia to an anti-inflammatory phenotype via FFAR2 in vitro

To verify the direct or indirect effects of *R.i.* on microglia, an in vitro model with lipopolysaccharide (LPS) and corticosterone (CORT) is used to simulate the stress and inflammatory state of depression. As depicted in Figure S2A-B, treatment with 1 µg/mL LPS and 0.1 µg/mL CORT significantly increased nitric oxide (NO) release and elevated the expression of pro-inflammatory cytokines (TNF-α, IL-1β and IL-6); reciprocally, it obviously reduced the level of TGF-β, without compromising cell viability, therefore, this dosage of LPS and CORT was chosen for subsequent experiments.

**Fig. 12:**
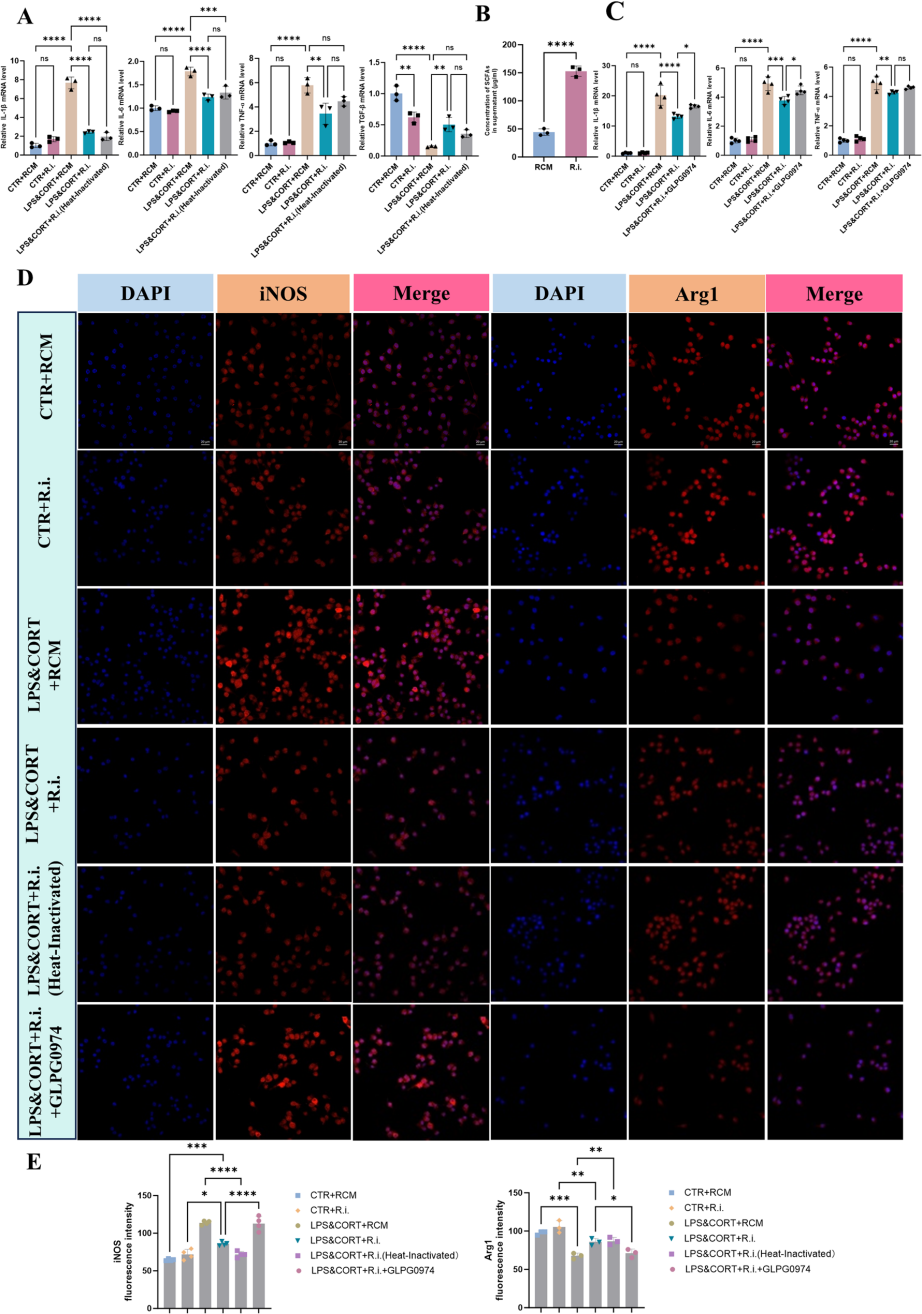
*R.i.*-conditioned medium modulates inflammatory cytokine expression and ameliorates microglial activation. **A** Expression levels of inflammatory cytokines in BV2 microglial cells following intervention with *R.i.*-conditioned medium. Data are from three independent biological replicates, each performed in triplicate. **B** Quantification of SCFA levels in *R.i.*-conditioned medium. Data are from three independent biological replicates, each performed in triplicate. **C** Alterations in inflammatory cytokine expression in BV2 cells. Data are from three independent biological replicates, each performed in quadruplicate. **D** Representative immunofluorescence images of the iNOS and Arg1 in microglial cells. Scale bar: 50 μm. **E** Quantitative analysis of immunofluorescence intensity. *n* = 4 for each group(iNOS), *n* = 3 for each group(Arg1). All data are presented as mean ± SEM. *p < 0.05, **p < 0.01, ***p < 0.001, ****p < 0.0001; ns, not significant

In dept to explore the salutary effects of *R.i.*, BV2 cells were divided into five treatment groups: CTR + RCM (regular culture medium), CTR + *R.i.* (*R.i.*-conditioned medium), LPS&CORT + RCM, LPS&CORT + *R.i.* (*R.i.*-conditioned medium), and LPS&CORT + *R.i.* (Heat-Inactivated). Analysis of inflammation-related gene comparing to the LPS&CORT + RCM group revealed that, both of the LPS&CORT + *R.i.* (*R.i.*-conditioned medium) and LPS&CORT + *R.i.* (Heat-Inactivated) groups displayed significantly reduced expression of pro-inflammatory cytokines (IL-1β, IL-6, TNF-α) and increased expression of the anti-inflammatory cytokine TGF-β (Fig. 12A), implying the important roles of the non-protein components in the supernatant in the anti-inflammatory response of *R.i.*. Having ruled out the protein-dependent effects and combined the properties of *R.i.* in SCFAs production, we verified the significant higher SCFA levels in *R.i.*-conditioned medium compared to sterile culture medium, confirming the SCFA specifically produced by *R.i.* (Fig. 12B). On the basis of this result, treatment with GLPG0974 partially abrogated the anti-inflammatory effects of *R.i.*-conditioned medium on inflamed BV2 cells. These observations suggest that *R.i.* produced SCFAs directly contribute to the suppression of neuroinflammation mediated by FFAR2 (Fig. 12C).

Microglia, responsive to environmental stimuli, can adopt distinct functional states: one characterized by the upregulation of pro-inflammatory cytokines and neurotoxic effects, and another that mitigates neuroinflammation and exerts neuroprotective functions [31]. Then we evaluated the polarization markers in BV2 cells following *R.i.*-conditioned medium intervention. LPS and CORT co-stimulation obviously enhanced the expression of inducible nitric oxide synthase (iNOS), a marker associated with the pro-inflammatory functional state, and decreased the expression of arginase-1 (Arg1), a marker associated with the anti-inflammatory and neuroprotective functional state. Notably, these pro-inflammatory polarization phenotypes were substantially reversed by the treatment with *R.i.*-conditioned medium. However, this ameliorative effect was completely abrogated upon inhibition of FFAR2, indicating that the *R.i.*-mediated shift in microglial polarization is dependent on the SCFA-FFAR2 signaling pathway (Fig. 12D-E).

## Discussion

Adolescent depression has emerged as an increasingly pressing global public health concern, with a multifactorial etiology encompassing genetic, environmental, psychological, and neurobiological factors. Unlike adult depression, this condition exhibits not only distinct pathophysiological profiles but also unique gut microbial signatures [32], a finding that furnishes critical clues for the present study. Our clinical investigation in female adolescents revealed that the abundance of the genus *Roseburia*, which harbor the capacity of short-chain fatty acids (SCFAs) production was significantly decreased in medication-free individuals with depressive adolescents (DEP) compared to Healthy controls (HC), implying the potential roles of SCFAs in modulation of the psychiatric disorder. Metagenomic sequencing has confirmed that *Roseburia* harbors complete gene sets encoding key enzymes for butyrate synthesis (e.g., butyryl-CoA transferase gene) [33], and the accuracy of this structural prediction was validated by the evidence of the current study that *Roseburia intestinalis (R.i.)* transplantation significantly impeded the CRS induced butyrate decrease, which is in line with the results that the abundance of *R.i.* is significantly positively correlated with fecal butyrate levels, and it exclusively produces butyrate via the fermentation of dietary fibers (such as inulin and pectin), without participating in the synthesis of other SCFAs like acetate or propionate [34]. Collectively, these lines of evidence establish a clear molecular basis for the direct regulatory role of *Roseburia* in modulating intestinal butyrate levels [35].

Moreover, a further correlation analysis between SCFA levels and Revised Child Anxiety and Depression Scale-25 (RCADS-25) depression scores revealed a significant negative correlation specifically between butyrate levels and depression severity. This finding directly supports our central hypothesis that reduced butyrate production is closely associated with the severity of depression in adolescents. Additionally, we observed a negative correlation between fecal acetic acid levels and RCADS scores. Previous research has indicated that members of the *Bacteroides* genus are capable of producing acetic acid [36], which is in line with a reduction in the abundance of fecal *Bacteroides* in patients with depression compared to healthy controls in the present work. These findings collectively suggest a potential link between acetic acid and depressive states. Research indicates that butyrate helps maintain intestinal barrier integrity and gut-brain axis homeostasis. Supplementation with butyrate has been shown to enhance cognitive function and memory, and to be involved in the pathophysiology of depression. These beneficial effects are achieved through several key mechanisms: alleviating microglia-mediated neuroinflammation, modulating the microbiota-gut-brain axis, and ameliorating damage within the central nervous system [37, 38]. Then we tie the reduced butyrate to the depletion of *Roseburia* in adolescent with the core assumption that butyrate produced by *Roseburia* may participate in and play prominent roles in maintaining immune homeostasis, a key mechanism in adolescent depression. To this end, we employed *R.i.* as an interventional strain in adolescent depression mice. To dissect *R.i.*’s potential role along the “gut-immune-brain” axis, we further designed two complementary experimental strategies using pharmacological and immunological assays: specific blockade of the butyrate receptor free fatty acid receptor 2 (FFAR2) and depletion of Treg cells. Behavioral assessments revealed that *R.i.* transplantation significantly ameliorated depression- and anxiety-like behaviors in CRS mice. Notably, this beneficial effect was strikingly abrogated when mice were co-administered GLPG0974. This “intervention-antagonism” experiment directly confirmed that *R.i.*’s antidepressant effect is dependent on FFAR2-mediated butyrate signaling: even with *R.i.* intervention, depressive behaviors persisted if the butyrate receptor was blocked.

Integrating behavioral and microbiota metabolic results, we demonstrated that the antidepressant effect of *R.i.* is essentially achieved through butyrate production and subsequent activation of the FFAR2, a mechanism consistent across both human and animal experiments.

Since the highly expression of FFAR2 in CD4^+^ T cells [39], and SCFAs, particularly butyrate, have been shown to influence central emotional states by modulating Treg cell function [40]. Although the recovery of Treg cell levels following *R.i.* intervention was found in the current study, the potential roles of Treg cells in the antidepressant process of *R.i.* transplantation remains unclear. Thus, an immunological assay of depletion of Treg cells by a CD25-neutralizing antibody (PC61.5) was exploited, intriguingly, the salutary effects of *R.i.* on depressive behaviors were completely abolished upon Treg depletion, deciphering for the first time, that Treg cells are essential effector cells in the antidepressant effects of *R.i.* transplantation.

The effects of SCFAs on immune cells have shown that butyrate promotes the expression of the transcription factor Foxp3, which is critical for maintaining the immunosuppressive function of Treg cells and serves as a key regulator for Treg cell differentiation [41]. Our cytokine profiling revealed that CRS mice exhibited decreased levels of anti-inflammatory factors and Foxp3, and reciprocally increased levels of pro-inflammatory factors levels, along with elevated microglial activation marker CD68 in the cortex, hippocampus, and colon. Noteworthily, *R.i.* intervention significantly reversed these inflammatory imbalances via Treg cells, since the anti-inflammatory effects of *R.i.* were completely eliminated upon Treg cell depletion. Thus, we propose that butyrate produced by *R.i.* promotes Treg cell differentiation and functional maintenance via activation of Foxp3 signaling, thereby increasing anti-inflammatory cytokine release, suppressing pro-inflammatory factor production, and ultimately alleviating central and peripheral inflammation.

However, in the clinical context, multiple research groups have reported distinct findings and outcomes in the field of depression research. two studies have revealed that in patients with major depressive disorder (MDD), the number of CD4⁺ CD25⁺ FOXP3⁺ regulatory T (Treg) cells is significantly increased, the population of CD4⁺ IL-10⁺ cells are markedly reduced, and a large amount of inflammatory factors are produced, which reveals that such inflammatory profiles are indicative of the progressive status of MDD. In light of this important observation, it is possible that the expansion of CD4⁺ CD25⁺ FOXP3⁺ Treg cells may reflect a crucial defensive mechanism for the body to persistently counteract the progression of depression, thereby leading to the coexistence of both anti-inflammatory and pro-inflammatory states [42, 43]. In our mouse model subjected to consecutive 28-day high-intensity stress stimulation, Treg cells not only displayed a notable functional decline—characterized by a significant reduction in IL-10 secretion—but also presented a distinct decrease in the number of CD4⁺ CD25⁺ FOXP3⁺ Treg cells. However, supplementation with *R.i.* effectively increased Treg cell counts and reversed depressive-like phenotypes in the mice. More importantly, depletion of Treg cells via antibody treatment almost abolished the protective effects of *R.i.*, which underscores the critical role of Treg cells in this process. Although the results derived from human subjects and the mouse model are not entirely consistent, the regulatory function of Treg cells in modulating depression is conserved across both MDD patients and depressed mice. The discrepancies between the two sets of results can be attributed to two main factors: first, the species differences between humans and mice; second, the fact that depression in humans is a gradually progressing pathological process, whereas in mice, it is a stress-induced condition with a relatively high-intensity progressive pattern.

A growing body of evidence suggests that impaired structure and function of microglia in the brain are implicated in the etiology of depression [44]. Alterations in microglia across different brain regions, including the prefrontal cortex (PFC), hippocampus (HIP), anterior cingulate cortex (ACC) and amygdala, have been linked to the depression development. Of note, the microglia undergo pronounced activation in the PFC and ACC during major depressive episodes [45]. In the present work, *R.i.* intervention significantly mitigated the CRS-induced microglial overactivation in the cortex and hippocampus, manifesting as the reduced cell numbers, decreasing soma size, and increasing dendritic branching and length. Meanwhile, neurogenesis, the process by which neural stem cells (NSCs) generate new neurons, promotes structural plasticity in the brain. The sequential expression of DCX and NeuN serves as a widely utilized molecular signature, marking the distinct stages of this process—from early progenitors to mature neurons [46, 47]. Research has revealed that chronic stress reduces the proliferation of newborn cells in the adult hippocampus [48]. Interestingly, the presence of new neurons is sufficient to maintain the antidepressant effects and reverse depressive phenotypes in mouse models [49, 50], which in line with the salutary effects of *R.i.* on restoring the number of neurons in the hippocampus. However, these improvements in microglial morphology and neurogenesis were completely abolished upon Treg cell depletion. These effects, at least to some extent reflect the essential roles of Treg cells, which serve as a bridge between gut microbiota and the brain.

However, how Treg cells regulates the microglia remains unknown in the current study, we suspected that the increased Treg cells stimulated by *R.i.* in depressive mice senses the gut inflammation and can be attracted to the damaged intestinal tract, where the Treg cells inhibit pro-inflammatory effects by release of anti-inflammatory cytokines such as IL-10 and TGF-β, which can cross the blood brain barrier [51]; another possibility is that microglia activation attracts a population of IL-10-producing Treg cells that migrated to the brain [52], thereby alleviating neuroinflammatory suppression of hippocampal NSC proliferation and ultimately facilitates neurogenesis, and the supposition is partially verified by the expression of the Foxp3 in the brain.

Moreover, the keys roles of FFAR2 in activation of Treg cells is also attractive in the present work, since blockage of this receptor obviously eliminated the beneficial effects of *R.i.* on microglia and neurogenesis, confirming that the “Treg cell-neural functional axis” is essentially a downstream extension of the “*R.i.*-butyrate-FFAR2” pathway, forming a complete “metabolite-immune-brain” regulatory cascade.

In the brain, microglia can adopt distinct functional states in response to environmental signals. These states are characterized by specific molecular markers and functional tendencies. One phenotype, often associated with the expression of molecules such as CD86 and inducible nitric oxide synthase (iNOS) [53, 54], exert pro-inflammatory effects, promote the synthesis of inflammatory mediators including TNF-α and IL-1β, and contribute to chronic neuroinflammation, phagocytosis, oxidative stress, and neurodegeneration [55], exhibiting neurotoxic properties [56]. Conversely, an alternative state, marked by the expression of molecules including CD206, CD163, and arginase-1 (Arg1), release neuroprotective and anti-inflammatory cytokines such as IL-10 and TGF-β, and facilitate neuroprotection and tissue repair, playing a neuroprotective role [57, 58]. To further specifically clarify the direct regulatory effect of *R.i.* on microglia, an LPS and corticosterone (CORT)-induced stress and inflammatory model in BV2 microglial cell was established. As expected, co-stimulation with LPS and CORT polarized microglia towards a pro-inflammatory functional state, manifested by elevated levels of pro-inflammatory factors and a corresponding decrease in anti-inflammatory factors. Interestingly, the *R.i.*-conditioned medium or even the heated *R.i.*-conditioned medium can efficaciously reverse the inflammatory outcomes, suggesting the beneficial components of the non-protein components, particularly the butyrate. Moreover, the specific contribution of butyrate to the *R.i.*-mediated shift in microglial functional state was confirmed, as pharmacological inhibition of FFAR2 significantly reversed the *R.i.*-mediated effects: the reduction in the pro-inflammatory marker iNOS and the enhancement of the anti-inflammatory marker Arg1 expression. These cellular-based results directly demonstrate that *R.i.* supernatant, via butyrate-mediated FFAR2 activation, inhibits the pro-inflammatory functional state of microglia and promotes their transition towards an anti-inflammatory and neuroprotective phenotype, thereby alleviating neuroinflammation. These results complement the in vivo findings, indicating that *R.i.* regulates microglial function through the butyrate-FFAR2-Treg cell pathway.

## Limitation

Although this study systematically elucidates the underlying mechanism by which *Roseburia* ameliorates adolescent depression and highlights the synergistic potential of “*R.i.*-butyrate-Treg cell-brain” axis, there are still some limitations: First, the clinical cohort included only female adolescents, and the sample size is small, and future studies should expand the sample size and include male participants; Second, the pharmacological inhibition of FFAR2 was not specific to Treg cells, and potential off-target effects in other tissues or cell types cannot be excluded; Third, it remains unclear whether the anti-neuroinflammatory effects of *R.i.* are mediated through the modulation of protective factor secretion by Treg cells or via Treg recruitment to the brain, further experimental validation is required to elucidate the precise mechanism. Finally, although all enrolled depressive patients were treatment-naïve, first-visit individuals, we did not adequately consider the potential influence of trained immunity on the subjects.

## Conclusion

**Fig. 13:**
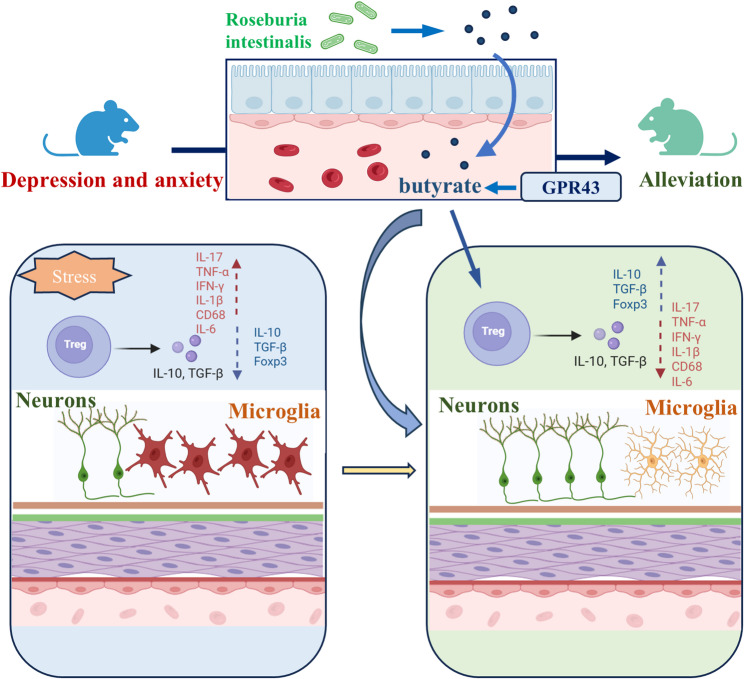
A schematic model summarizes the proposed mechanism. A reduction in *R.i.* abundance leads to decreased SCFA production, with butyrate serving as a key effector. This decline in SCFAs impairs the ability of the SCFA receptor FFAR2 to promote the proliferation and differentiation of regulatory T (Treg) cells, disrupting the balance between pro-inflammatory and anti-inflammatory responses. Over time, this sustained immune imbalance triggers central neuroinflammation, which ultimately facilitates the emergence of depression-like phenotypes. In contrast, *R.i.* transplantation reverses this pathological cascade: it restores butyrate production, thereby activating the butyrate-FFAR2 signaling axis. This signaling activation enhances Treg cell expansion, which in turn mitigates neuroinflammation and normalizes depression-like behaviors

In summary, this study reveals an unrecognized the pivotal roles of *R.i.* in adolescent depression, pinpointing the intricate crosstalk across the *R.i.*, its metabolite (butyrate), Treg cells and microglia. The depletion of *R.i.* and the resultant decreased butyrate contributes to the declined Treg activity and expanding via FFAR2, thereby, disrupting the balance between pro-inflammatory and anti-inflammatory responses. and triggering neuroinflammation and impairing hippocampal neurogenesis-two key pathological processes that ultimately drive the development of depression in adolescents. Thus, strategies targeting this “*R.i.*-butyrate-Treg-microglia” axis, including *R.i.* transplantation, butyrate supplementation, or Treg cell activation, hold significant promise as novel therapeutic approaches for adolescent depression (Fig. 13).

## Supplementary Information

Supplementary Material 1.

## Data Availability

All data and materials associated with this study are present in the paper or the Supplementary Materials.

## References

[1] Shorey S, Ng ED, Wong CHJ. Global prevalence of depression and elevated depressive symptoms among adolescents: A systematic review and meta-analysis. Br J Clin Psychol. 2022;61(2):287–305. 34569066 10.1111/bjc.12333

[2] Zwolińska W, Dmitrzak-Węglarz M, Słopień A. Biomarkers in Child and Adolescent Depression. Child Psychiatry Hum Dev. 2023;54(1):266–81. 34590201 10.1007/s10578-021-01246-y PMC9867683

[3] Zhou J, Liu Y, Ma J, Feng Z, Hu J, Hu J, et al. Prevalence of depressive symptoms among children and adolescents in china: a systematic review and meta-analysis. Child Adolesc Psychiatry Ment Health. 2024;18(1):150. 39563377 10.1186/s13034-024-00841-w PMC11577650

[4] Flores MW, Sharp A, Carson NJ, Cook BL. Estimates of Major Depressive Disorder and Treatment Among Adolescents by Race and Ethnicity. JAMA Pediatr. 2023;177(11):1215–23. 37812424 10.1001/jamapediatrics.2023.3996 PMC10562990

[5] Avenevoli S, Swendsen J, He JP, Burstein M, Merikangas KR. Major depression in the national comorbidity survey-adolescent supplement: prevalence, correlates, and treatment. J Am Acad Child Adolesc Psychiatry. 2015;54(1):37–e442. 25524788 10.1016/j.jaac.2014.10.010 PMC4408277

[6] Liu L, Wang H, Chen X, Zhang Y, Zhang H, Xie P. Gut microbiota and its metabolites in depression: from pathogenesis to treatment. EBioMedicine. 2023;90:104527. 36963238 10.1016/j.ebiom.2023.104527 PMC10051028

[7] Socała K, Doboszewska U, Szopa A, Serefko A, Włodarczyk M, Zielińska A, et al. The role of microbiota-gut-brain axis in neuropsychiatric and neurological disorders. Pharmacol Res. 2021;172:105840. 34450312 10.1016/j.phrs.2021.105840

[8] Radford-Smith DE, Anthony DC. Prebiotic and Probiotic Modulation of the Microbiota-Gut-Brain Axis in Depression. Nutrients. 2023;15(8):1880. 37111100 10.3390/nu15081880 PMC10146605

[9] Liang S, Wu X, Hu X, Wang T, Jin F. Recognizing Depression from the Microbiota⁻Gut⁻Brain Axis. Int J Mol Sci. 2018;19(6):1592. 29843470 10.3390/ijms19061592 PMC6032096

[10] Kovtun AS, Averina OV, Angelova IY, Yunes RA, Zorkina YA, Morozova AY, et al. Alterations of the Composition and Neurometabolic Profile of Human Gut Microbiota in Major Depressive Disorder. Biomedicines. 2022;10(9):2162. 36140263 10.3390/biomedicines10092162 PMC9496097

[11] Nie K, Ma K, Luo W, Shen Z, Yang Z, Xiao M, et al. Roseburia intestinalis: A Beneficial Gut Organism From the Discoveries in Genus and Species. Front Cell Infect Microbiol. 2021;11:757718. 34881193 10.3389/fcimb.2021.757718 PMC8647967

[12] Zhao M, Ren Z, Zhao A, Tang Y, Kuang J, Li M et al. Gut bacteria-driven homovanillic acid alleviates depression by modulating synaptic integrity. Cell Metab. 2024;36(5):1000-12.e6. 10.1016/j.cmet.2024.03.010 38582087

[13] Zhou M, Fan Y, Xu L, Yu Z, Wang S, Xu H, et al. Microbiome and tryptophan metabolomics analysis in adolescent depression: roles of the gut microbiota in the regulation of tryptophan-derived neurotransmitters and behaviors in human and mice. Microbiome. 2023;11(1):145. 37386523 10.1186/s40168-023-01589-9 PMC10311725

[14] Zhao C, Bao L, Qiu M, Wu K, Zhao Y, Feng L, et al. Commensal cow Roseburia reduces gut-dysbiosis-induced mastitis through inhibiting bacterial translocation by producing butyrate in mice. Cell Rep. 2022;41(8):111681. 36417859 10.1016/j.celrep.2022.111681

[15] Dong J, Wang B, Xiao Y, Liu J, Wang Q, Xiao H, et al. Roseburia intestinalis sensitizes colorectal cancer to radiotherapy through the butyrate/OR51E1/RALB axis. Cell Rep. 2024;43(3):113846. 38412097 10.1016/j.celrep.2024.113846

[16] Kang X, Liu C, Ding Y, Ni Y, Ji F, Lau HCH, et al. Roseburia intestinalis generated butyrate boosts anti-PD-1 efficacy in colorectal cancer by activating cytotoxic CD8(+) T cells. Gut. 2023;72(11):2112–22. 37491158 10.1136/gutjnl-2023-330291 PMC10579466

[17] Hao F, Tian M, Zhang X, Jin X, Jiang Y, Sun X, et al. Butyrate enhances CPT1A activity to promote fatty acid oxidation and iTreg differentiation. Proc Natl Acad Sci U S A. 2021;118:22. 10.1073/pnas.2014681118 PMC8179238 34035164

[18] Guo TT, Zhang Z, Sun Y, Zhu RY, Wang FX, Ma LJ et al. Neuroprotective Effects of Sodium Butyrate by Restoring Gut Microbiota and Inhibiting TLR4 Signaling in Mice with MPTP-Induced Parkinson’s Disease. Nutrients. 2023;15(4). 10.3390/nu15040930 PMC9960062 36839287

[19] Fock E, Parnova R. Mechanisms of Blood-Brain Barrier Protection by Microbiota-Derived Short-Chain Fatty Acids. Cells. 2023;12(4):657. 36831324 10.3390/cells12040657 PMC9954192

[20] Li Y, Liu A, Chen K, Li L, Zhang X, Zou F, et al. Sodium butyrate alleviates lead-induced neuroinflammation and improves cognitive and memory impairment through the ACSS2/H3K9ac/BDNF pathway. Environ Int. 2024;184:108479. 38340407 10.1016/j.envint.2024.108479

[21] Wei H, Yu C, Zhang C, Ren Y, Guo L, Wang T, et al. Butyrate ameliorates chronic alcoholic central nervous damage by suppressing microglia-mediated neuroinflammation and modulating the microbiome-gut-brain axis. Biomed Pharmacother. 2023;160:114308. 36709599 10.1016/j.biopha.2023.114308

[22] Stilling RM, van de Wouw M, Clarke G, Stanton C, Dinan TG, Cryan JF. The neuropharmacology of butyrate: The bread and butter of the microbiota-gut-brain axis? Neurochem Int. 2016;99:110–32. 27346602 10.1016/j.neuint.2016.06.011

[23] Furusawa Y, Obata Y, Fukuda S, Endo TA, Nakato G, Takahashi D, et al. Commensal microbe-derived butyrate induces the differentiation of colonic regulatory T cells. Nature. 2013;504(7480):446–50. 24226770 10.1038/nature12721

[24] Spies G, Stein DJ, Roos A, Faure SC, Mostert J, Seedat S, et al. Validity of the Kessler 10 (K-10) in detecting DSM-IV defined mood and anxiety disorders among pregnant women. Arch Womens Ment Health. 2009;12(2):69–74. 19238521 10.1007/s00737-009-0050-0

[25] Deng Y, Zhou M, Wang J, Yao J, Yu J, Liu W, et al. Involvement of the microbiota-gut-brain axis in chronic restraint stress: disturbances of the kynurenine metabolic pathway in both the gut and brain. Gut Microbes. 2021;13(1):1–16. 33535879 10.1080/19490976.2020.1869501 PMC7872056

[26] Papouin T, Haydon PG. Obtaining Acute Brain Slices. Bio Protoc. 2018;8(2). 10.21769/BioProtoc.2699 PMC5856250 29552595

[27] Zhou L, Wang C, Gao J, Wu X, Li G, Jiang X, et al. Novel Role of Gut-Derived Roseburia Intestinalis in Safeguarding Intestinal Barrier Integrity and Microenvironment Homeostasis During Arsenic Exposure. Adv Sci (Weinh). 2025;12(42):e11895. 40831218 10.1002/advs.202511895 PMC12622465

[28] Xing Y, Wang M, Yuan Y, Hu J, Wang Z, Sun Z, et al. Gut microbiota-derived butyrate mediates the anticolitic effect of indigo supplementation through regulating CD4(+) T cell differentiation. Imeta. 2025;4(3):e70040. 40469515 10.1002/imt2.70040 PMC12130576

[29] Wang Q, Hu Y, Li F, Hu L, Zhang Y, Qiao Y, et al. MgSO(4) alleviates hippocampal neuroinflammation and BBB damage to resist CMS-induced depression. Front Nutr. 2025;12:1470505. 40206943 10.3389/fnut.2025.1470505 PMC11979798

[30] Zhao J, Zhang M, Zhang H, Wang Y, Chen B, Shao J. Diosmin ameliorates LPS-induced depression-like behaviors in mice: Inhibition of inflammation and oxidative stress in the prefrontal cortex. Brain Res Bull. 2024;206:110843. 38092305 10.1016/j.brainresbull.2023.110843

[31] Paolicelli RC, Sierra A, Stevens B, Tremblay ME, Aguzzi A, Ajami B, et al. Microglia states and nomenclature: A field at its crossroads. Neuron. 2022;110(21):3458–83. 36327895 10.1016/j.neuron.2022.10.020 PMC9999291

[32] Tian T, Qin Y, Wu M, Wang W, Song T, Deng X, et al. Differential gut microbiota and microbial metabolites in adolescents with depression. Asian J Psychiatr. 2023;83:103496. 36764124 10.1016/j.ajp.2023.103496

[33] Vital M, Howe AC, Tiedje JM. Revealing the bacterial butyrate synthesis pathways by analyzing (meta)genomic data. mBio. 2014;5(2):e00889. 24757212 10.1128/mBio.00889-14 PMC3994512

[34] Louis P, Hold GL, Flint HJ. The gut microbiota, bacterial metabolites and colorectal cancer. Nat Rev Microbiol. 2014;12(10):661–72. 25198138 10.1038/nrmicro3344

[35] Song WS, Jo SH, Lee JS, Kwon JE, Park JH, Kim YR, et al. Multiomics analysis reveals the biological effects of live Roseburia intestinalis as a high-butyrate-producing bacterium in human intestinal epithelial cells. Biotechnol J. 2023;18(12):e2300180. 37596881 10.1002/biot.202300180

[36] Makki K, Deehan EC, Walter J, Bäckhed F. The Impact of Dietary Fiber on Gut Microbiota in Host Health and Disease. Cell Host Microbe. 2018;23(6):705–15. 29902436 10.1016/j.chom.2018.05.012

[37] Cheng J, Hu H, Ju Y, Liu J, Wang M, Liu B, et al. Gut microbiota-derived short-chain fatty acids and depression: deep insight into biological mechanisms and potential applications. Gen Psychiatr. 2024;37(1):e101374. 38390241 10.1136/gpsych-2023-101374 PMC10882305

[38] Cristiano C, Cuozzo M, Coretti L, Liguori FM, Cimmino F, Turco L, et al. Oral sodium butyrate supplementation ameliorates paclitaxel-induced behavioral and intestinal dysfunction. Biomed Pharmacother. 2022;153:113528. 36076609 10.1016/j.biopha.2022.113528

[39] Huang S, Hu S, Liu S, Tang B, Liu Y, Tang L, et al. Lithium carbonate alleviates colon inflammation through modulating gut microbiota and Treg cells in a GPR43-dependent manner. Pharmacol Res. 2022;175:105992. 34801681 10.1016/j.phrs.2021.105992

[40] Merchak A, Gaultier A. Microbial metabolites and immune regulation: New targets for major depressive disorder. Brain Behav Immun Health. 2020;9:100169. 34589904 10.1016/j.bbih.2020.100169 PMC8474524

[41] Lv J, Hao P, Zhou Y, Liu T, Wang L, Song C, et al. Role of the intestinal flora-immunity axis in the pathogenesis of rheumatoid arthritis-mechanisms regulating short-chain fatty acids and Th17/Treg homeostasis. Mol Biol Rep. 2025;52(1):617. 40544212 10.1007/s11033-025-10714-w

[42] Daray FM, Grendas LN, Arena ÁR, Tifner V, Álvarez Casiani RI, Olaviaga A, et al. Decoding the inflammatory signature of the major depressive episode: insights from peripheral immunophenotyping in active and remitted condition, a case-control study. Transl Psychiatry. 2024;14(1):254. 38866753 10.1038/s41398-024-02902-2 PMC11169351

[43] Tifner V, Daray FM, López-Carvajal JE, Arena ÁR, Grendas LN, Álvarez Casiani RI, et al. Unbalanced CD4(+)IL-10(+) and more activated and exhausted CD8(+) lymphocytes characterized patients with a major depressive episode. J Pharmacol Exp Ther. 2025;392(5):103563. 40215833 10.1016/j.jpet.2025.103563

[44] Jia X, Gao Z, Hu H. Microglia in depression: current perspectives. Sci China Life Sci. 2021;64(6):911–25. 33068286 10.1007/s11427-020-1815-6

[45] Wang H, He Y, Sun Z, Ren S, Liu M, Wang G, et al. Microglia in depression: an overview of microglia in the pathogenesis and treatment of depression. J Neuroinflammation. 2022;19(1):132. 35668399 10.1186/s12974-022-02492-0 PMC9168645

[46] Masanetz RK, Mundlos H, Stolzer I, Winkler J, Günther C, Süß P. Absence of Microglial Activation and Maintained Hippocampal Neurogenesis in a Transgenic Mouse Model of Crohn’s Disease. Cells. 2025;14(11). 10.3390/cells14110841 PMC12155506 40498017

[47] Huang H, Liu CM, Sun J, Hao T, Xu CM, Wang D, et al. Ketamine Affects the Neurogenesis of the Hippocampal Dentate Gyrus in 7-Day-Old Rats. Neurotox Res. 2016;30(2):185–98. 26966008 10.1007/s12640-016-9615-7

[48] Zhang SQ, Deng Q, Zhu Q, Hu ZL, Long LH, Wu PF, et al. Cell type-specific NRBF2 orchestrates autophagic flux and adult hippocampal neurogenesis in chronic stress-induced depression. Cell Discov. 2023;9(1):90. 37644025 10.1038/s41421-023-00583-7 PMC10465581

[49] Tunc-Ozcan E, Peng CY, Zhu Y, Dunlop SR, Contractor A, Kessler JA. Activating newborn neurons suppresses depression and anxiety-like behaviors. Nat Commun. 2019;10(1):3768. 31434877 10.1038/s41467-019-11641-8 PMC6704083

[50] Abbott LC, Nigussie F. Adult neurogenesis in the mammalian dentate gyrus. Anat Histol Embryol. 2020;49(1):3–16. 31568602 10.1111/ahe.12496

[51] White Z, Cabrera I, Mei L, Clevenger M, Ochoa-Raya A, Kapustka I, et al. Gut inflammation promotes microbiota-specific CD4 T cell-mediated neuroinflammation. Nature. 2025;643(8071):509–18. 40533562 10.1038/s41586-025-09120-w

[52] Izzy S, Yahya T, Albastaki O, Abou-El-Hassan H, Aronchik M, Cao T, et al. Nasal anti-CD3 monoclonal antibody ameliorates traumatic brain injury, enhances microglial phagocytosis and reduces neuroinflammation via IL-10-dependent T(reg)-microglia crosstalk. Nat Neurosci. 2025;28(3):499–516. 40016353 10.1038/s41593-025-01877-7 PMC11893472

[53] Lisi L, Ciotti GM, Braun D, Kalinin S, Currò D, Dello Russo C, et al. Expression of iNOS, CD163 and ARG-1 taken as M1 and M2 markers of microglial polarization in human glioblastoma and the surrounding normal parenchyma. Neurosci Lett. 2017;645:106–12. 28259657 10.1016/j.neulet.2017.02.076

[54] Zhou T, Huang Z, Sun X, Zhu X, Zhou L, Li M, et al. Microglia Polarization with M1/M2 Phenotype Changes in rd1 Mouse Model of Retinal Degeneration. Front Neuroanat. 2017;11:77. 28928639 10.3389/fnana.2017.00077 PMC5591873

[55] Orihuela R, McPherson CA, Harry GJ. Microglial M1/M2 polarization and metabolic states. Br J Pharmacol. 2016;173(4):649–65. 25800044 10.1111/bph.13139 PMC4742299

[56] Kwon HS, Koh SH. Neuroinflammation in neurodegenerative disorders: the roles of microglia and astrocytes. Transl Neurodegener. 2020;9(1):42. 33239064 10.1186/s40035-020-00221-2 PMC7689983

[57] Liu LR, Liu JC, Bao JS, Bai QQ, Wang GQ. Interaction of Microglia and Astrocytes in the Neurovascular Unit. Front Immunol. 2020;11:1024. 32733433 10.3389/fimmu.2020.01024 PMC7362712

[58] Tang Y, Le W. Differential Roles of M1 and M2 Microglia in Neurodegenerative Diseases. Mol Neurobiol. 2016;53(2):1181–94. 25598354 10.1007/s12035-014-9070-5

