# Supplementary material for: Roseburia intestinalis ameliorates adolescent depression via GPR43‑dependent Treg cell expansion and suppression of neuroinflammation
Source: J Neuroinflammation. 2026 Mar 3;23:113. doi: 10.1186/s12974-026-03755-w (PMC13063914; doi:10.1186/s12974-026-03755-w)
Supplement: Supplementary file 1 — Supplementary Material 1. [file 12974_2026_3755_MOESM1_ESM.doc]

Supplementary Materials for

***Roseburia intestinalis* ameliorates adolescent depression via GPR43‑dependent Treg cell expansion and suppression of neuroinflammation**

# Supplemental Methods

## 1. Forced Swim Test (FST)

The forced swim test was performed in a transparent glass tank, with a video camera positioned in front for behavioral recording. Each mouse was individually assessed by placing it in the tank filled with water maintained at 23±1°C. The swimming behavior was recorded for a total duration of 6 minutes. Immobility was defined as the absence of active movement, except for minimal motions required to maintain the head above water. The total immobility time was measured, and its proportion relative to the test duration was calculated. Behavioral analysis was conducted using SuperFst software (Soft-Maze Software Co., China).

## 2. Tail Suspension Test (TST)

In the tail suspension test, each mouse was individually assessed by securing its tail with adhesive tape 3 cm from the tip and suspending it in a head-down position approximately 15 cm above the surface. Initially, mice exhibit active escape behaviors to counteract the unnatural posture, but over time, they display intermittent immobility, which is indicative of behavioral despair. The test lasted for 6 minutes, during which the total immobility time was recorded. Following the test, mice were promptly removed and returned to their home cages. The immobility ratio was quantified using Tail Suspension-Scan software (Geneandi Co., China) throughout the observation period to evaluate behavioral despair.

## 3. Elevated Plus Maze (EPM)

The elevated plus maze comprises two open arms, two closed arms, and a central zone. Each mouse was placed in the central area facing an open arm, and its behavior was recorded for 6 minutes using a video tracking system. The duration spent in both the open and closed arms was measured. To prevent olfactory interference in subsequent trials, fecal matter was removed after each test, and the maze was disinfected with 75% ethanol and wiped with a clean cloth. The locomotor activity and exploratory behavior of the mice were recorded and analyzed using ANY-maze software (Stoelting Co., USA).

## 4. Open Field Test (OFT)

The open field test was conducted in a square arena with a camera positioned overhead for behavioral tracking. The floor of the arena was divided into a central zone and a peripheral zone. Each mouse was placed in the central area, and its movement was recorded for 6 minutes. The number of crossings into the central area and the total duration spent in this region were measured. To eliminate residual odors and prevent olfactory interference, feces were removed after each test, and the floor was disinfected with 75% ethanol and wiped with a clean cloth. The time spent in different zones was recorded and analyzed using ANY-maze software (Stoelting Co., USA).

# Table S1. Demographic characteristics of depressive and healthy control participants.

| Characteristic | HC (n = 10) | DEP (n = 25) | *p* value |
| --- | --- | --- | --- |
| *Adolescent characteristics* | | | |
| Age (years) | 14.80±0.42 | 13.76 ±1.23 | *p*=0.014* |
| Body weight (kg) | 53.08±2.63 | 57.10±2.55 | *p*=0.365 |
| BMI | 21.02±1.03 | 21.42±0.94 | *p*=0.811 |
| *Parental characteristics* | | | |
| *Marital status (n (%))* | | | |
| Married | 10 (100) | 18 (72) | *p*=0.084 |
| Separated or divorced or widowed | 0 (0) | 7 (28) |
| *Paternal education level (n (%))* | | | |
| ≤junior high school | 5 (50) | 20 (80) | *p*=0.107 |
| ＞junior high school | 5 (50) | 5 (20) |
| *Paternal employment status (n (%))* | | | |
| Employed | 8 (80) | 15 (60) | *p*=0.434 |
| Unemployed | 2 (20) | 10 (40) |
| *Maternal education level (n (%))* | | | |
| ≤junior high school | 7 (70) | 18 (72) | *p*=1.000 |
| ＞junior high school | 3 (30) | 7 (28) |
| *Maternal employment status (n (%))* | | | |
| Employed | 9 (90) | 16 (64) | *p*=0.218 |
| Unemployed | 1 (10) | 9 (36) |
| *Gestational characteristics* | | | |
| Parity (n (%)) | | | |
| 1 | 10 (100) | 21 (84) | *p*=1.000 |
| ≥2 | 0 (0) | 3 (16) |
| Pregnant age | 24.60±2.76 | 25.96±4.57 | *p*=0.388 |
| Emotional state of pregnancy (n (%)) | | | |
| Fine | 10 (100) | 23 (92) | *p*=1.000 |
| Depressive | 0 (0) | 2 (8) |

Data are presented as the mean ± SEM. ^*^p＜0.05 versus the HC group. Except for age, body weight and BMI differences between HC and DEP groups tested by Student’s *t*-test, other features were analyzed by chi-square test or Fisher’s exact test.

# Table S2. RCADS-25 Scores in Adolescent Females with Depression

| **Number** | 1 | 2 | 3 | 4 | 5 | 6 | 7 | 8 | 9 | 10 | 11 | 12 | 13 | 14 | 15 | 16 | 17 | 18 | 19 | 20 |
| --- | --- | --- | --- | --- | --- | --- | --- | --- | --- | --- | --- | --- | --- | --- | --- | --- | --- | --- | --- | --- |
| **Total RCADS score** | 61 | 49 | 74 | 77 | 67 | 70 | 57 | 78 | 74 | 76 | 85 | 85 | 83 | 80 | 84 | 90 | 86 | 82 | 70 | 59 |

# Table S3. Primers used for RT-qPCR

| ﻿Target Gene | ﻿Primer Sequences (5’-3’) | |
| --- | --- | --- |
|  | Forward | Reverse |
| *Gapdh* | GTGGACCTCATGGCCTACAT | TGTGAGGGAGATGCTCAGTG |
| *IFN-γ* | AGCGGCTGACTGAACTCAGATTGTAG | GTCACAGTTTTCAGCTGTATAGGG |
| *Foxp3* | CCCAGGAAAGACAGCAACCTT | TTCTCACAACCAGGCCACTTG |
| *IL-17* | CTCCAGAAGGCCCTCAGACTAC | AGCTTTCCCTCCGCATTGACAC |
| *IL-6* | ACCGCTATGAAGTTCCTCTC | CTCTGTGAAGTCTCCTCTCC |
| *IL-10* | GCTCTTACTGACTGGCATGAG | CGCAGCTCTAGGAGCATGTG |
| *TGF-β* | GCAACATGTGGAACTCTACCAGAA | GACGTCAAAAGACAGCCACTCA |
| *CD68* | CCACAGGCAGCACAGTGGACA | TCCACAGCAGAAGCTTTGGCCC |
| *TNF-α* | TCTATGGCCCAGACCCTCAC | GACGGCAGAGAGGAGGTTGA |
| *IL-1β* | AAGGTCCACGGGAAAGACAC | AGCTTCAGGCAGGCAGTATC |
| PCR① | CTTATCAGTGGGTGGAGCAATAAG | CCTTGCTATTTCAGCATGGTG |
| PCR② | CAATGGAGCTGTTTGGATGGTAC | TGCCTTATTCCCACTGAACCTG |

# Supplemental Figures

**
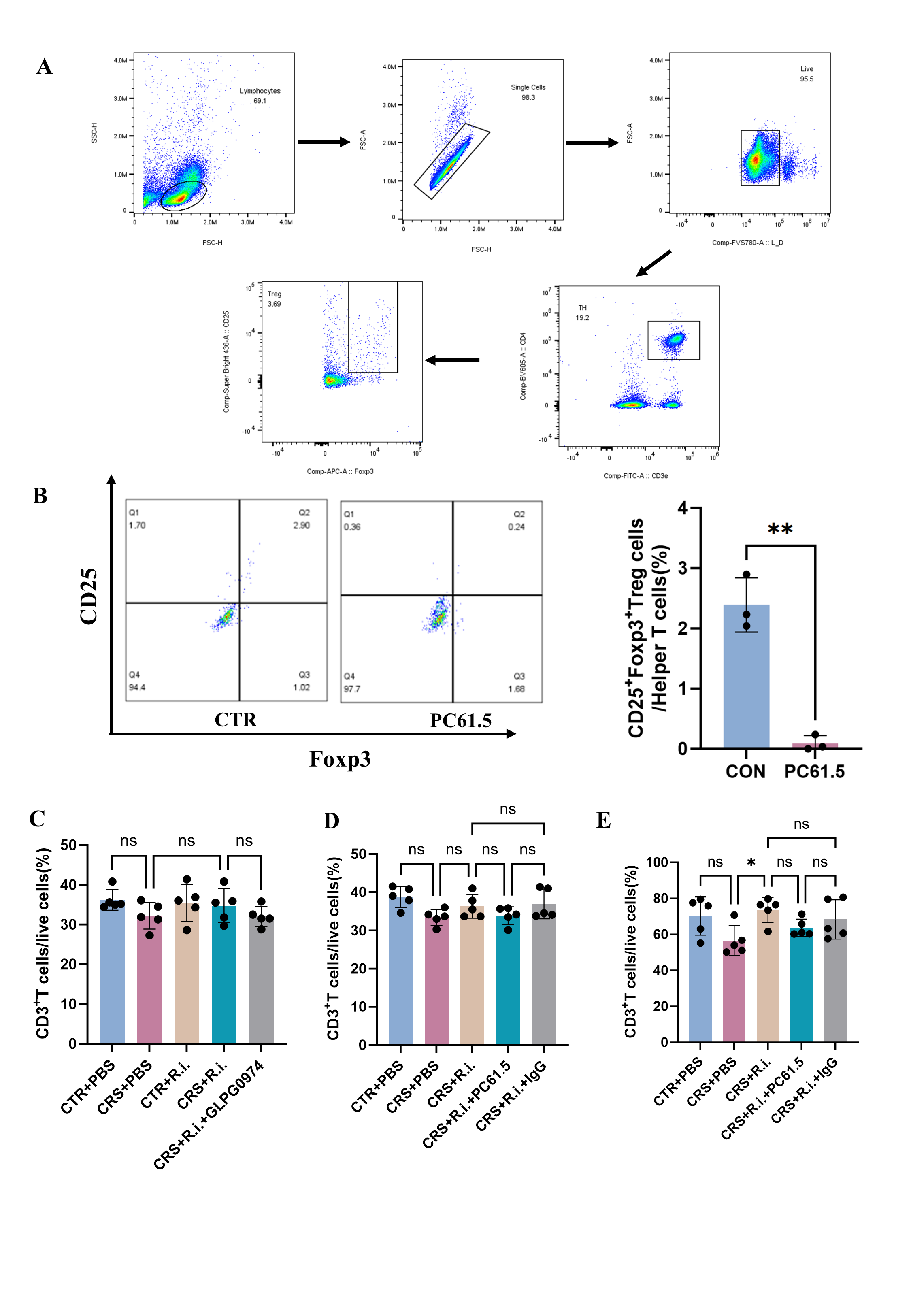
Figure S1.** **Gating Strategy for Flow Cytometry and Treg Cell Depletion Analysis** (A) Gating strategy for flow cytometric analysis of T cells. (B) Relative reduction in regulatory T (Treg) cell levels in blood on day 14 after PC61.5 injection compared to IgG control. (C) Quantification of CD3^+^ T cell levels in the blood of CRS mice following *R.i.* intervention. (D)Quantification of CD3^+^ T cell levels in the blood of CRS mice following *R.i.* intervention after Treg depletion. (E)Quantification of CD3^+^ T cell levels in the colon of CRS mice following *R.i.* intervention after Treg depletion. All data are presented as mean ± SD. *p < 0.05, **p < 0.01; ns, no significance.


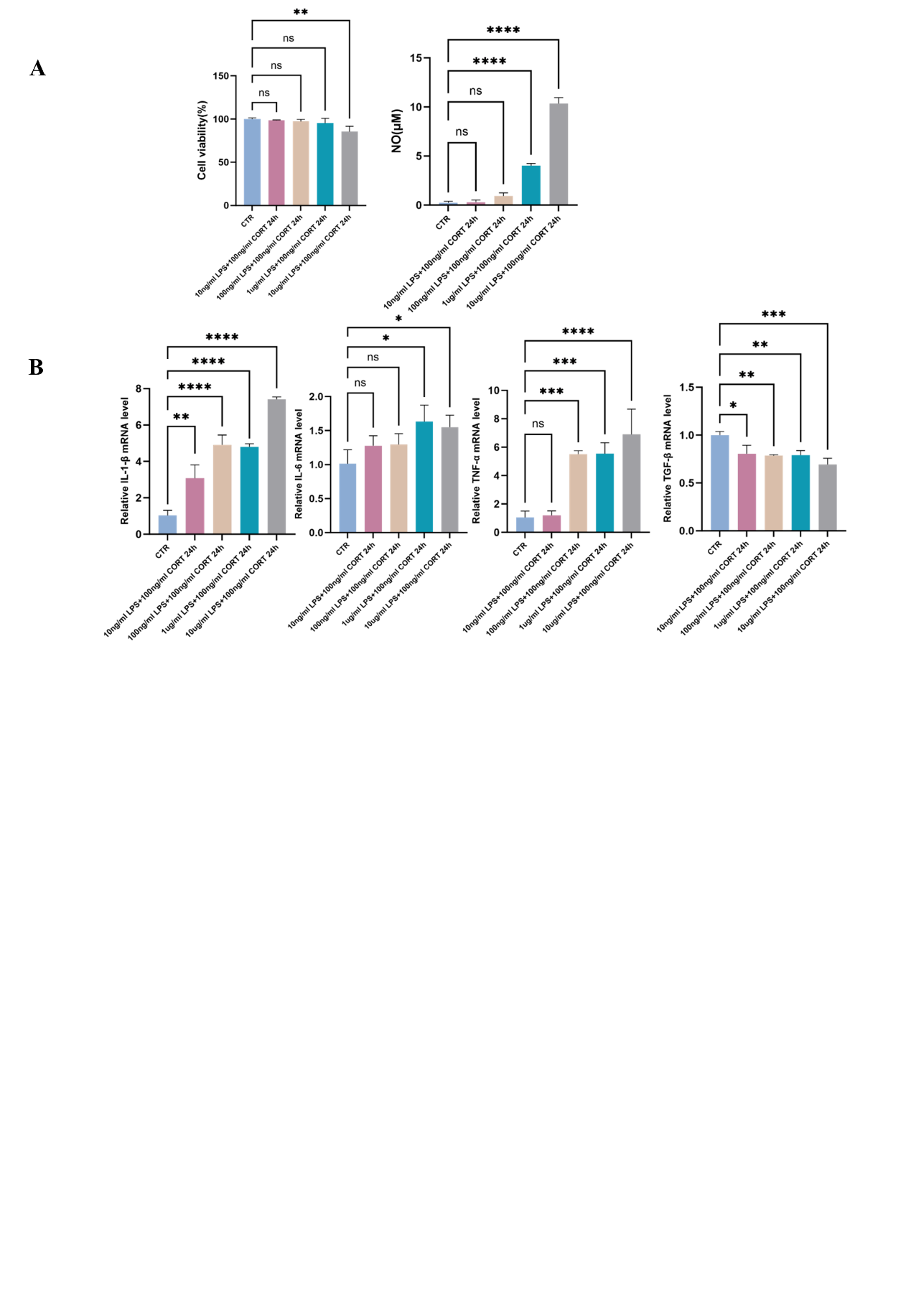
 **Figure S2.** **Establishment of the LPS + CORT Cellular Model.** (A) CCK-8 assay results for cells treated with a gradient concentration of LPS.  Nitric oxide (NO) expression levels in cells treated with a gradient concentration of LPS. (B) Expression levels of inflammatory cytokines in cells treated with a gradient concentration of LPS. All data are presented as mean ± SEM. *p < 0.05, **p < 0.01, ***p < 0.001, and ****p < 0.0001; ns, no significance.
